# The mechanisms of manual therapy: A living review of systematic, narrative, and scoping reviews

**DOI:** 10.1371/journal.pone.0319586

**Published:** 2025-03-18

**Authors:** Damian L. Keter, Joel E. Bialosky, Kevin Brochetti, Carol A. Courtney, Martha Funabashi, Steve Karas, Kenneth Learman, Chad E. Cook

**Affiliations:** 1 Physical Medicine and Rehabilitation Department, United States Department of Veterans Affairs, Cleveland, Ohio, United States of America; 2 Department of Physical Therapy, University of Florida, Gainesville, Florida, United States of America; 3 Brooks-PHHP Research Collaboration, Gainesville, Florida, United States of America; 4 Department of Physical Therapy and Human Movement Sciences, Northwestern University, Chicago Illinois, United States of America; 5 Division of Research and Innovation, Canadian Memorial Chiropractic College, Toronto, Canada; 6 Department of Chiropractic, Université du Québec à Trois-Rivières, Trois-Rivières, Canada; 7 Research Center, Parker University, Dallas, Texas, United States of America; 8 Department of Physical Therapy, Chatham University, Pittsburgh, Pennsylvania, United States of America; 9 Department of Graduate Studies in Health and Rehabilitation Sciences, Youngstown State University, Youngstown, Ohio, United States of America; 10 Department of Orthopaedics, Duke University, Durham, North Carolina, United States of America; 11 Department of Population Health Sciences, Duke University, Durham, North Carolina, United States of America; 12 Duke Clinical Research Institute, Duke University, Durham, North Carolina, United States of America; University of Würzburg, GERMANY

## Abstract

**Introduction:**

Treatment mechanisms are the underlying process or pathway through which a treatment influences the body. This includes molecular, cellular and physiological processes or pathways contributing to treatment effect. Manual therapy (MT) evokes complex mechanistic responses across body systems, interacting with the individual patient and context to promote a treatment response. Challenges arise as mechanistic studies are spread across multiple professions, settings and populations. The purpose of this review is to summarize treatment mechanisms that have been reported to occur with MT application.

**Methods:**

Four electronic databases were searched (Medline, CINAHL, Cochrane Library, and PEDro) for reviews investigating mechanistic responses which occur during/post application of MT. This review was registered *a priori* with PROSPERO (CRD42023444839). Methodological quality (AMSTAR-2) and risk of bias (ROBIS) were assessed for systematic and scoping reviews. Data were synthesized by mechanistic domain.

**Results:**

Sixty-two reviews were included. Systematic reviews (n = 35), narrative reviews (n = 24), and scoping reviews (n = 4) of asymptomatic (n = 37), symptomatic (n = 43), non-specified human subjects (n = 7) and animals (n = 7) were included. Reviews of moderate quality supported neurovascular, neurological, and neurotransmitter/neuropeptide changes. Reviews of low quality supported neuroimmunce, neuromuscular, and neuroendocrine changes. Reviews of critically low quality support biomechanical changes.

**Conclusions:**

Findings support critically low to moderate quality evidence of complex multisystem mechanistic responses occurring with the application of MT. Results support peripheral, segmental spinal, and supraspinal mechanisms occurring with the application of MT, which can be measured directly or indirectly. The clinical value of these findings has not been well established. While MT has proven to be an effective intervention to treat conditions such as pain, the current body of literature leaves uncertainty as to ‘why’ MT interventions work, and future research should look to better define which mechanisms (or combinations of mechanisms) are mediators of clinical response.

## Introduction

Manual Therapy (MT) is a type of force-based manipulation which has been defined as *“passive application of mechanical force to the outside of the body with therapeutic intent, often as part of pain management care (e.g., low-back pain), rehabilitation care, or general wellness and disease prevention”* [1]. Techniques associated with MT include soft tissue mobilization (STM), joint mobilization (non-thrust), and manipulation (thrust). These techniques are used by healthcare professionals such as osteopaths, massage therapists, chiropractors, and physical therapists (PT). Whereas historical models of MT attributed the clinical effect to biomechanical changes within tissues directly related to the technique applied [2,3], recent evidence-based models support more complex interactive mechanistic responses across body systems, interacting with the individual patient and context to promote a treatment response [4–6].

Mechanisms are “*the molecular, cellular, physiological processes or pathways contributing to a) disease development, b) treatment action, or c) pain signal sensation, transmission, perception, and modulation”* [7]. Treatment mechanisms are “*the underlying process or pathway through which a specific treatment produces an influence on the body”* [7]. Potential treatment mechanisms associated with MT include: biomechanical (altered tissue movement, fluid loading, etc.), neurological (fMRI activation, altered neural conduction, etc.), neuroimmune (release of inflammatory and anti-inflammatory mediators, etc.), neurovascular (sympathetic response, etc.), neurotransmitter and neuropeptide (release of serotonin, beta endorphin, etc.), neuromuscular (altered neuromuscular tone, muscle recruitment), neuroendocrine (release of cortisol etc.) and other mechanisms [8].

Mechanisms are processes that can be measured in a number of different ways. Measurements can be direct or indirect. Higher level (cortical and subcortical) changes typically represent initial efferent activity (direct measure), while measurements of downstream effects represent indirect or proxy measures of the process (e.g., skin conductance as a proxy measure of ANS response, fMRI as proxy measure of cortical activation, neuroimmune markers as proxy measures of multisystem subcortical activation, somatosensory reflexes as a measure of spinal excitability, etc.) rather than direct/primary measures (e.g., EEG as a measure of cortical activity, MRI as a measure of joint position, etc.) [9]. Defined mechanistic domains and proposed direct and indirect measures within these domains are presented in S1 Appendix. Studies investigating treatment mechanisms associated with MT are of interest as recent National Institute of Health (NIH) funding has been designated to better understand the mechanisms associated with the application of force during MT [1].

MT techniques have demonstrated efficacy/effectiveness in improving range of motion, reducing disability, improving function and modulating pain [10–12]. Based on these findings, the use of selected MT techniques are commonly cited in high-level clinical practice guidelines as recommended interventions for various conditions [13–17]. Despite this endorsement, variability exists in the strength of recommendations [18,19], suggesting an inconclusive body of literature. Furthermore, reported treatment effect sizes for MT are small to moderate [20], which is likely due to individual variability in treatment response [21] when provided with a one-size-fits-all treatment approach [22]. Mechanistic-based treatment stratification represents a potential approach to match patients to treatments and improve outcomes [21,23,24]. Such an approach allows the matching of an intervention of known mechanisms to patients with underlying conditions responsive to these mechanisms [25]. Clarifying the mechanisms through which MT inhibits musculoskeletal pain could improve the effectiveness in clinical practice by better informing a mechanistic approach to identifying individuals who will respond positively to these interventions. Despite progress in this area of study the mechanisms underpinning the demonstrated effectiveness of MT remains unclear due to two notable limitations:

1) Mechanism-based studies are spread across multiple professions and settings including lab-based and clinical-based designs, animal and human models, and asymptomatic and symptomatic specimens.
2) Mechanism-based studies often lack translation of causality between treatment mechanisms and clinical outcomes (translational studies).

A recent interprofessional panel reached a consensus on current gaps that are present in mechanistic research [8]; however, the literature involves multiple physiological systems, often explored heterogeneously, making it challenging to interpret and summarize. The purpose of this review of reviews was to identify and summarize the neurological, neuroimmune, biomechanical, neurovascular, neurotransmitter/neuropeptide, neuroendocrine, and other not previously categorized treatment mechanism systems that have been reported to occur with MT application. Reviews of reviews play a crucial role in synthesizing and evaluating existing research, providing a comprehensive perspective on a specific topic or question therefore aiding both researchers and clinicians to make sense of a vast complex topic [26].

## Methods

### Protocol and registration

A review of systematic, scoping, and narrative reviews was performed to assess and summarize the mechanisms associated with MT application. To encompass new evidence as it becomes available, a living review building off the findings of this study will be hosted digitally at the Duke Center for Excellence in Manual and Manipulative Therapy (CEMMT). This study was registered with PROSPERO on September 03, 2023, prior to the initial literature search (CRD42023444839).

### Eligibility criteria

Systematic reviews, scoping reviews, and narrative reviews with or without meta-analyses were included. Reviews including MT techniques within the scope of Physical Therapy (PT) practice were included (mobilization (non-thrust), manipulation (thrust), STM/massage, light touch). Manual techniques controlled or performed by external, non-human forces were excluded except for instrument-assisted STM in which a human provider was manually controlling a device to assist in external tissue mobilization. Internal (invasive) STM techniques such as dry needling and acupuncture were excluded. Outcomes (mechanisms) required for inclusion were neurological, neuroimmune, biomechanical, neurovascular, neurotransmitter, neuroendocrine, and other non-aforementioned mechanisms associated with MT application. In vivo models including living human and animal subjects were included. In-vitro models and cadaveric studies were excluded as the treatment mechanisms are assumed to differ in these models. Comparators included control, sham, or other MT procedures. Reviews were excluded if they did not include an outlined literature search strategy.

### Information sources

Four electronic databases were searched including: Medline, Cumulative Index to Nursing and Allied Health Literature (CINAHL), Cochrane Library, and Physiotherapy Evidence Database (PEDro). Reviews published from inception to October 3, 2023 were included in initial search. An updated search was performed September 23, 2024 to include reviews published through that date. The search strategy for each of the included databases was validated by a Health Sciences librarian and uploaded to PROSPERO prior to the search. The comprehensive search strategy is available in S2 Appendix.

### Data selection

Two review authors performed title (DK, KB), abstract (DK, MF), and full text (DK, KB) screenings independently. Discrepancies between reviewers were resolved by a third review author (CC, KB). Microsoft Excel (Microsoft Corporation; version 2205-2211) was utilized to manage and organize the search results throughout the review process. Cohen’s Kappa Scores (95% CI) were calculated to assess agreement between reviewers.

### Data extraction

Data were extracted using a self-developed tool to extract appropriate variables. The following items were extracted: author, year, type of review, database(s) searched, number of included studies, mechanistic domain, MT intervention, comparator, outcome and results/conclusion. Data were extracted independently by two review authors (DK, KB) with all discrepancies discussed and agreed upon by the two authors.

### Methodological quality appraisal

Methodological quality for each included systematic and scoping review was performed using the AMSTAR-2 quality assessment measure [27]. Risk of Bias was assessed using the ROBIS tool [28]. Two review authors independently performed quality appraisal (KL, CC) and risk of bias assessment (SK, JB) with discrepancies resolved by a third review author (DK). Quality assessment and risk of bias was not assessed for narrative reviews.

### Data synthesis

Studies were grouped by mechanistic domains (S1 Appendix) outlined in previous work [8] with mechanisms outside of those domains categorized within an ‘other’ category. No synthesized quality or GRADE was used given the focus on mechanistic outcomes rather than clinical effectiveness.

### Data analysis

No analysis of data occurred. The goals of this review were to outline and summarize the status of the available literature.

## Results

### Selection of sources of evidence

**Fig 1:**
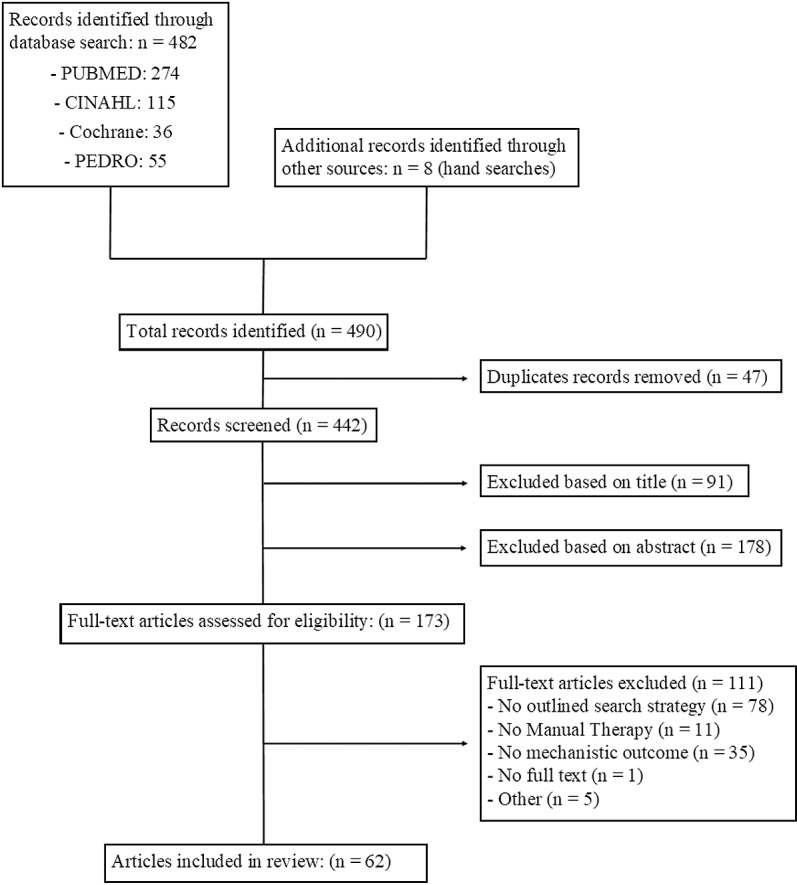
PRISMA Flowchart for selection of sources of evidence.

The search identified 442 reviews after duplicate removal. After title and abstract screening, 173 full-text reviews were agreed to be assessed for eligibility. Cohens Kappa coefficients to assess agreement between reviewers demonstrated moderate to strong agreement for title (*k* = .77; 95% CI.69 -.84) abstract (*k* = .83; 95% CI.78 -.89), and full text (*k* = .98; 95% CI.94 -.99) screening. Sixty-two reviews were agreed upon to be included in this review of reviews. A flowchart representing the process for evidence selection including rationale for full texts which were screened and excluded is presented in Fig 1. Detailed rationale for full texts which were screened and excluded are presented in S3 Appendix.

### Study characteristics

**Fig 2:**
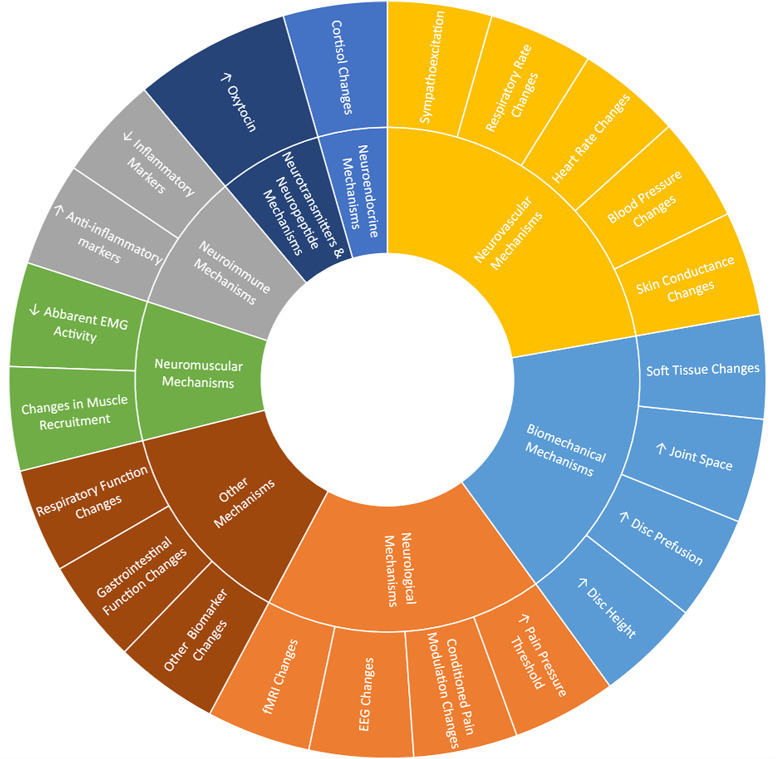
Treatment mechanisms occurring with Manual Therapy application.

Included reviews were systematic reviews (n = 35), narrative reviews (n = 24), and scoping reviews (n = 4). Databases searched within these reviews included CINAHL (n = 35), Cochrane (n = 27), PubMed/Medline (n = 61), PEDro (n = 17), SCOPUS (n = 10), and EMBASE (n = 19). Forty-five of the included reviews also included other search strategies (e.g., searching references of included studies, consulting experts in the field) and databases (PsycINFO, AMED, MANTIS, Google Scholar, Elsevier, Science Direct, Web of Science, Index of Chiropractic Literature, SciELO, etc.). Subjects included asymptomatic humans (n = 37), symptomatic humans (n = 43), human subjects without specifications regarding status (n = 7) and animal models (n = 6). MT techniques included manipulation (n = 41), mobilization (n = 23), and STM or massage (n = 19). Reviews included studies comparing MT to sham intervention (n = 37), comparing MT to control (no intervention) (n = 45), and 14 reviews that did not specify a comparator. Treatment mechanisms from the biomechanical domain (n =  14), neurovascular domain (n = 32), neurological domain (n = 23), neurotransmitter/neuropeptide domain (n = 16), neuroimmune domain (n = 12), neuroendocrine domain (n = 11), neuromuscular domain (n = 10), and other domains (n = 7) were identified (Fig 2). Characteristics of the included reviews are presented in Table 1 with more detailed extracted data presented in S4 Appendix. Reported treatment mechanisms across the aforementioned domains are presented in Tables 2–11.

**Table 1:** Characteristics of included reviews.

Author Year	Type of Review	Included Database(s)	(n) Studies	MT Technique(s)			Comparator(s)	Mechanistic Domain(s)
				Man	Mob	STM		
Alanazi et al. 2024 [29]	Narr. Review	Medline	221	Spinal, Peripheral	----------	----------	Sham, Control	Neurovascular
Araujo et al. 2019 [30]	Sys. Review	Cochrane, Medline, PEDro, Embase, Other	18	Spinal	Spinal	----------	Sham, Control	Neurovascular, Neurotransmitter
Arribas-Romano et al. 2020 [31]	Sys. Review	Cinahl, Cochrane, Medline, PEDro, SCOPUS, Embase, Other	17	Lumbar	Knee	----------	Sham, Control	Neurological
Bernier Carney et al. 2020 [32]	Sys. Review	Cinahl, Medline, SCOPUS, Other	2	-----------	----------	Gua sha	n/a	Neuroimmune, Neuroendocrine
Bolton et al. 2012 [33]	Narr. Review	Medline, Other	35	Spinal	Spinal	----------	Sham, Control	Neurovascular, Neuromuscular, Neuroimmune, Neuroendocrine, Neurotransmitter, Neuropeptide, Other
Borges et al. 2018 [34]	Sys. Review	Cochrane, Medline, PEDro, SCOPUS, Other	9	Spinal	----------	LE, UE, Lumbar, Craniofacial, Craniosacral	Sham, Control	Neurovascular
Chow et al. 2021 [35]	Sys. Review	Cinahl, Cochrane, Medline, Embase, Other	8	Spinal	----------	----------	Sham, Control	Neuroimmune, Neuroendocrine, Neurotransmitter, Neuropeptide, Neurovascular
Chu et al. 2014 [36]	Sys. Review	Cinahl, Cochrane, Medline, PEDro, Other	11	----------	Spinal	----------	Sham, Control	Neurovascular
Colombi et al. 2019 [37]	Narr. Review	Medline, Other	13	Spinal	----------	----------	Sham, Control	Neuroendocrine, Neuroimmune
Cook et al. 2024 [38]	Narr. Review	Medline	13	Spinal, Peripheral	Spinal, Peripheral	n/a	Sham, Control	Neuroimmune
Coronado et al. 2012[39]	Sys. Review	Cinahl, Medline, Other	20	Spinal	----------	----------	Sham, Control	Neurological
Coronado et al. 2013 [40]	Sys. Review	Cinahl, Medline, PEDro, Other	5	Spinal	----------	----------	Sham	Neurological, Neurotransmitter, Neuroendocrine
Corso et al. 2019 [41]	Sys. Review	Cinahl, Cochrane, PEDro Other	20	Spinal	----------	----------	Sham, Control	Neuromuscular, Neurovascular
Evans 2002 [42]	Narr. Review	Medline, Embase	n/a	Spinal	----------	----------	n/a	Biomechanical
Field 2016 [43]	Narr. Review	Medline	65	----------	----------	n/a	Sham	Neuroimmune, Neuroendocrine, Neuropeptide, Biomechanical, Neurovascular, Other
Galindez-Ibarbengoetxea et al. 2017 [44]	Sys. Review	Cinahl, Medline, PEDro, SCOPUS, Other	11	Cervical	----------	----------	Control	Neurovascular
Gay et al. 2005 [45]	Narr. Review	Cinahl, Medline, Embase, Other		Lumbar	----------	----------	n/a	Biomechanical
Gay et al. 2013 [46]	Sys. Review	Cinahl, Medline	23	----------	----------	n/a	Sham, Control	Neurological
Gera et al. 2020 [47]	Sys. Review	Cochrane, Medline, Other	18	Spinal	Spinal	----------	Sham, Control	Neurovascular
Haavik et al. 2021 (2) [48]	Narr. Review	Cinahl, Medline, Other	58	Spinal	----------	----------	n/a	Neuromuscular
Haavik et al. 2021 [49]	Narr. Review	Cinahl, Medline, SCOPUS, Other	23	Spinal	----------	----------	Control	Neuroimmune, Neuropeptide, Neuroendocrine, Neurotransmitter
Hartnett 2021[50]	Narr. Review	Medline, Other	57	----------	----------	n/a	Control	Biomechanical
Hegedus et al. 2011 [51]	Sys. Review	Cinahl, Medline, Other	10	----------	Spinal	----------	Control	Neurovascular
Hennenhoefer et al. 2019 [52]	Narr. Review	Medline, Other	69	Spinal	----------	----------	n/a	Biomechanical, Neuromuscular, Neurological
Hillier et al. 2010 [53]	Sys. Review	Cinahl, Cochrane, Medline, Embase, Other	4	----------	----------	n/a	Sham	Neuroimmune
Holey et al. 2014 [54]	Narr. Review	Cinahl, Medline, Other	n/a	----------	----------	n/a	n/a	Neurovascular, Neurotransmitter
Honoré et al. 2018 [55]	Sys. Review	Cochrane, Medline, Embase, Other	12	Spinal	----------	----------	Sham, Control	Neurological
Jacobson 2011 [56]	Narr. Review	Medline, Other	12	----------	----------	n/a	n/a	Neurological, Biomechanical, Neuromuscular, Other
Jones et al. 2013 [57]	Sys. Review	Cinahl, Cochrane, Medline, Embase, Other	12	----------	----------	Foot	Sham., Control	Neurovascular
Jun et al. 2020 [58]	Scop Review	Cinahl, Medline	10	Lumbar	Lumbar	----------	Control	Neuromuscular, Biomechanical, Neurological
Jung et al. 2023 [59]	Sys. Review	Cinahl, Cochrane, Medline, Other	22	Spinal	Spinal	----------	Sham, Control	Neurological
Kingston et al. 2014 [60]	Sys. Review	Cochrane, Medline, PEDro, Embase, Other	7	----------	Spinal	----------	Sham, Control	Neurovascular
Lascurain-Aguirrebena et al. 2016 [61]	Sys. Review	Cinahl, Medline, SCOPUS, Embase, Other	24	----------	Spinal	----------	Sham, Control	Neurological, Neurovascular, Biomechanical
Lima et al. 2020 [62]	Scop Review	Cinahl, Cochrane, Medline, Embase, Other	78	Spinal	Spinal, LE	n/a	n/a	Neuroimmune, Neuromuscular, Neurotransmitter, Neurological, Neurovascular, Biomechanical
Meyer et al. 2019 [63]	Sys. Review	Medline, PEDro, Embase	18	Spinal	----------	----------	Sham, Control	Neurological
Millan et al. 2012 [64]	Sys. Review	Cochrane, Medline, Other	43	Spinal	----------	----------	Sham, Control	Neurological
Mitchell et al. 2017 [65]	Sys. Review	Cinahl, Cochrane, Medline, SCOPUS, Other	8	Lumbar	Lumbar	----------	Sham, Control	Biomechanical
Moyer et al. 2011 [66]	Narr. Review	Cinahl, Medline, Other	19	----------	----------	Full Body	Control	Neuroendocrine
Navarro-Santana et al. 2020 [67]	Sys. Review	Cinahl, Cochrane, Medline, PEDro, SCOPUS, Embase, Other	17	----------	Spinal, UE	----------	Sham, Control	Neurovascular
Nelson 2015 [68]	Scop Review	Cinahl, Medline	27	----------	----------	n/a	n/a	Neurological, Neurovascular, Neuroendocrine
Picchiottino et al. 2019 [69]	Sys. Review	Cochrane, Medline, PEDro, Embase, Other	29	Spinal	Spinal Elbow	----------	Sham	Neurovascular, Neurotransmitter
Pickar 2002 [70]	Narr. Review	Medline, Other	n/a	Spinal	----------	----------	Sham, Control	Neuromuscular, Biomechanical
Potter et al. 2005 [71]	Narr. Review	Cinahl, Medline, Other	31	Spinal, Peripheral	----------	----------	n/a	Biomechanical, Neurological, Neurovascular, Neuromuscular, Neurotransmitter
Riley et al. 2024 [72]	Sys. Review	Cinahl, Medline, PEDro, Other	3	Spinal	----------	----------	Sham	Neurological
Rogan et al. 2023 [73]	Scop Review	Cochrane, Medline, PEDro, Other	33	Spinal	Spinal	Spinal	Sham, Control	Neurovascular
Sampath et al. 2017 [74]	Sys. Review	Cinahl, Cochrane, Medline, PEDro, SCOPUS, Embase, Other	8	Spinal	----------	----------	Sham, Control	Neuroimmune, Neuroendocrine, Neurotransmitter, Neuropeptide
Sampath et al. 2024 [75]	Sys. Review	Cinahl, Cochrane, Medline, SCOPUS, Other	14	Spinal	----------	----------	Sham, Control	Neurovascular, Neuropeptide/Neurotransmitter, Neuroendocrine
Savva et al. 2021 [76]	Narr. Review	Cinahl, Medline, Other	16	Spinal, UE	----------	----------	Sham Control	Neurological, Neurovascular
Schmid et al. 2008 [77]	Sys. Review	Cinahl, Cochrane, Medline	15	----------	Cervical	----------	Sham	Neuromuscular, Neurological, Neurovascular
Simmonds et al. 2012 [78]	Narr. Review	Cinahl, Medline. Other	13	Spinal	Spinal	----------	Sham, Control	Neurovascular
Sousa et al. 2020 [79]	Sys. Review	Medline, Other	10	Spinal	----------	Hand, Spinal	Sham, Control	Neurovascular
Sullivan et al. 2020 [80]	Sys. Review	Cinahl, Medline, Other	28	Spinal	----------	----------	Sham, Control	Neurovascular
Tejero-Fernandez et al. 2015 [81]	Sys. Review	Cochrane, Medline, PEDro	5	----------	----------	n/a	Control	Neuroimmune, Neurological, Neuroendocrine, Other
Vernon 2000 [82]	Narr. Review	Medline	11	Lumbar	----------	----------	Control	Neurological
Vicenzino 2007 [83]	Narr. Review	Cinahl, Cochrane, Medline, Embase, Other	19	----------	UE	----------	n/a	Biomechanical, Neurological, Neurovascular
Vigotsky et al. 2015 [84]	Narr. Review	Medline	29	Spinal, Knee	----------	----------	Sham, Control	Neurotransmitter, Neuropeptide
Voogt et al. 2015 [85]	Sys. Review	Cochrane, Medline, Embase, Other	14	Cervical	LE, UE, Cervical	----------	n/a	Neurological
Weerapong et al. 2005 [86]	Narr. Review	Medline, Other	35	----------	----------	Whole body	Control	Neurovascular
Xiong et al. 2015 [87]	Sys. Review	Cochrane, Medline, Embase, Other	24	----------	----------	n/a	Control	Neurovascular
Yao et al. 2014 [88]	Narr. Review	Medline	6	----------	Thorax	----------	Sham	Neurovascular, Neuroimmune
Young et al. 2024 [89]	Sys. Review	Cinahl, Cochrane, Medline, PEDro, Embase, Other	20	Spinal	----------	----------	Control	Biomechanical
Zegarra-Parodi et al. 2015 [90]	Sys. Review	Cinahl, Cochrane, Medline, PEDro	20	Spinal	Spinal	----------	Sham, Control	Neurovascular

Definitions: LE- Lower extremity, UE- Upper Extremity, n/a- not specified.

**Table 2:** biomechanical treatment mechanisms associated with MT.

	Author/Year	Subjects	MT Technique	Measure	Time of Measure	Results/Conclusion
Systematic Reviews	Lascurain-Aguirrebena et al. 2016 [61]	Humans- Neurogenic UE pain, LBP, neck pain, craniofacial pain, shoulder pain, cervicogenic dizziness, whiplash, epicondylalgia	Mob- Spinal	Imaging	During and Immediately post to 6 weeks post	Lumbar: PA mobilization produced intersegmental extension at each targeted segment, greatest at L2-L3 and least at L4-L5 Cervical: PA mobilization only produced compression of the soft tissue; no intersegmental motion at any cervical joint Variability in post PA motion: 1 favoring changes in motion and 1 reporting no significant difference
	Mitchell et al. 2017 [65]	Humans- Not specified	Man- Lumbar Mob- Lumbar	Disc height, morphology, molecular transportation, intervertebral disc space and diffusion	n/a	All studies confirmed, either directly or indirectly, that their respective intervention influenced disc physiology primarily through water flow
	Young et al. 2024 [89]	Humans- Healthy, LBP, non-migraine headaches Animals- Dogs, horses	Man- Spinal	Radiography, Ultrasound, Stiffness assessment	Immediate Post	Change in vertebral position in 2/3 included studies (0/2 supporting rated credible) ↑ Facet joint space in 4/5 included studies (all 4 supporting rated credible) Changes in spinal stiffness in 3/3 included studies (1/3 supporting rated credible) Changes in muscle stiffness in 2/6 included studies (0/2 supporting rated credible) Changes in disc pressure in 1/1 included study rated as not credible Changes in tissue characteristics in 1/1 included study rated as not credible Changes in tissue damage to artery in 0/1 included study rated as not credible
Scoping Reviews	Lima et al. 2020 [62]	Animals- Non-Cadaveric Animal Models (Rats, Rabbits, Mice); healthy and induced pain	STM- Region not specified	Imaging	n/a	↓ Myofibril damage → Muscle volume ↑ Cross sectional area ↑ Force production
	Jun et al. 2020 [58]	Humans- Asymptomatic and symptomatic; Animals	Man- Lumbar Mob- Lumbar	Dorsoventral displacement, Intervertebral disc diffusion	n/a	Six studies suggested that changes in spinal or bending stiffness are associated with an increase in mobility following SMT Insufficient fluid recovery resulting in a change in the viscoelastic properties of the soft tissues Repeated loading of the spine might cause creep and relaxation of the spinal connective tissues, which would change the resistance to the applied load and similarly the initial displacement under the applied load ↑ Intervertebral disc diffusion following SMT, but only in the participants classified as responders at follow-up
Narrative Reviews	Jacobson 2011 [56]	Humans- Asymptomatic	STM- Structural integration		n/a	None of the hypotheses about the local effects of structural integration manipulation have been assessed
	Hennenhoefer et al. 2019 [52]	Humans- Not specified	Man- Region not specified	Joint position	n/a	Correcting the asymmetrical movement preferences of vertebrae (or even affecting the vertebrae at all) is unlikely to be a source of therapeutic benefit
	Hartnett 2021 [50]	Humans- Symptomatic	STM- Region not specified	Scar tissue breakdown, tissue perfusion	n/a	Proposed mechanisms include combination of increased tissue perfusion and scar tissue breakdown- minimal strong evidence supporting the proposed physiological effects of the therapy
	Potter et al. 2005 [71]	Humans- Not specified	Man- Spinal and Peripheral	Joint space on MRI	Immediate Post	↑ Joint space at MCP pre vs post ↑ Joint space at spine (facet joints) pre vs post and vs control
	Field 2016 [43]	Humans- Patients with burn scar	STM- Region not specified	Imaging	n/a	↓ Scar tissue
	Evans 2002 [42]	Humans- Not specified	Man- Spinal	Imaging (MRI)	Immediate post	Transient relative movements of the manipulated vertebrae associated with cavitation Gapping of zygapophyseal joints ↑ Dimensions of the intervertebral foramen
	Vicenzino 2007 [83]	Humans- Post-traumatic thumb injury	Mob (MWM)- MCP	Imaging (MRI)	n/a	MRI revealed 4 deg pronated positional fault of MCP joint before treatment, which was not present with MWM
	Pickar 2002 [70]	Humans- Asymptomatic and symptomatic	Man- Spinal		n/a	↑ Joint space (MCP and Lumbar Facet)
	Gay et al. 2005 [45]	Humans- Not specified	Man- Lumbar	CT- Disc Height, Degree of disc protrusion, Central and lateral canal space; Intradiscal pressure	Immediate post	↑ Disc height ↓ Disc protrusion/abnormality → Percent of the canal occupied by the disc pre vs post-treatment ↑ Intradiscal pressure in all cases except rotation with distraction, which resulted in very little change or a decrease in pressure

Definitions: ↑- Increase, ↓- Decrease, →- No change, STM- Soft tissue mobilizations, Man- Manipulation, Mob- Mobilizations, PA- Posterior to anterior, UE- Upper extremity, LBP- Low back pain, MCP- Metacarpophalangeal, MWM- Mobilization with movement, SMT- Spinal manipulative technique.

**Table 3:** Neurovascular treatment mechanisms associated with MT.

	Author/Year	Subjects:	MT Technique	Measure	Time of Measure	Results/Conclusion
Systematic Reviews	Corso et al. 2019 [41]	Humans- Asymptomatic	Man- Spinal	HR, HRV	Immediate post	→ Resting HR or HRV → Exercising HR
	Kingston et al. 2014 [60]	Humans- Asymptomatic and symptomatic (neck pain)	Mob- Spinal	ST, SC, RR, BP, HR	During to immediate post	Strong evidence for sympathoexcitation vs control and sham: ↑ SC ↑ RR ↑ BP ↑ HR ↓ ST
	Hegedus et al. 2011 [51]	Humans- Asymptomatic and symptomatic (neck pain, lateral elbow pain)	Man- Spinal	α -amylase secretion (saliva), SC, ST	Immediate to 24 hrs post	↑ SC in healthy subjects (lasting 10 minutes max and 5 minutes or less avg.) → ST in healthy subjects ↓ α -amylase (lasting 10 minutes)
	Gera et al. 2020 [47]	Humans- Asymptomatic and symptomatic (LBP, Neck Pain, Thoracic spine pain, HTN)	Man- Spinal Mob- Spinal	BP, HR, ECG, Pulse-ox	n/a	↓ SBP (MD = 4:56, 95% CI = 9:20, 0.08; p = .05) ↓ DBP vs control and vs placebo group (MD = 1:96, 95% CI = 4:60, 0.69; p = 0:15) Changes in HR (direction not specified) (MD = 0:24, 95%CI = 3:59, 3.11; p = 0:89)
	Zegarra-Parodi et al. 2015 [90]	Humans- Asymptomatic and symptomatic (Low Back Pain, Lateral epicondylalgia, Neck pain)	Man- Spinal Mob- Spinal, SI	SC, ST, laser doppler flowmetry (LDF)	n/a	Healthy populations: ↑ SC ↓ ST ↓ LDF among smokers and ↑ LDF among nonsmokers Symptomatic populations: ↑ SC in individuals with cervical and craniofacial pain ↓ SC in individuals with LBP ↓ ST in individuals with cervical pain or epicondylalgia ↑ ST in individuals with LBP ↓ LDF in individuals with epicondylalgia
	Chow et al. 2021 [35]	Humans- Asymptomatic and symptomatic	Man- Spinal	HRV	n/a	→ HRV
	Sullivan et al. 2020 [80]	Humans- Not specified	Man- Spinal	BP	Immediate	13 studies did not report any significant changes in blood pressure and 14 studies showed significant changes in blood pressure after chiropractic intervention Cervical spine SMT more consistently demonstrate ↓ BP (Sympathetic inhibitory)
	Galindez-Ibarbengoetxea et al. 2017 [44]	Humans- Asymptomatic and symptomatic (HTN)	Man- Cervical	BP, HR, ECG, Pulse-ox	Immediate to post 8-sessions	↓ DBP → HR, SBP, ECG, Pulse-Ox
	Araujo et al. 2019 [30]	Humans- Asymptomatic and symptomatic (craniofacial pain, neck pain)	Man- Spinal Mob- Spinal	HR, RR, BP, SC, pupil	n/a	PAIVM mobilization: ↑ SC and ↓ ST compared to no treatment in patients with neck pain (low quality evidence) ↑ SC and ↓ ST in patients with neck pain compared with placebo (low quality evidence) ↑ HR in healthy individuals (low quality evidence) ↑ SC compared to placebo in healthy individuals (low quality evidence) ↑ SC in patients with craniofacial pain (low quality evidence) ↑ HR vs placebo (low quality evidence) ↑ HR in patients with craniofacial pain (low quality evidence) → ST vs placebo or control in healthy individuals (very low quality evidence) → ST compared to placebo (low quality evidence) SNAG mobilizations: → SC in healthy individuals (low quality evidence) → ST vs placebo in healthy individuals (very low quality evidence) Manipulation: ↓ Edge light pupil cycle time in healthy participants (very low quality evidence) → Pupil diameter (low quality evidence)
	Sousa et al. 2020 [79]	Humans- Asymptomatic and symptomatic (back pain, fibromyalgia)	Man- Thoracic and cervical STM- Hand, paravertebral	Cardiac Autonomic control (heartrate variability)	Immediate to 20 weeks	9/10 studies demonstrated improvement in cardiac autonomic control. The only study that did not observe improvement in cardiac autonomic control was performed on patients with fibromyalgia.
	Navarro-Santana et al. 2020 [67]	Humans- Asymptomatic and symptomatic (Elbow pain, neck pain, craniofacial pain)	Mob- Spinal	ST, SC	During to immediate post	↑ SC (SMD 1.21, 95% CI 0.88 to 1.53, n = 269) vs control ↑ SC (SMD 0.73, 95% CI 0.51to 0.96, n = 293) vs sham ↓ ST (SMD 0.92, 95% CI − 1.47 to − 0.37, n = 128) vs control ↓ ST (SMD − 0.50, 95% CI − 0.82 to − 0.18, n = 134) vs sham
	Borges et al. 2018 [34]	Humans- Asymptomatic and symptomatic (fibromyalgia)	Man- Spinal STM- LE’s, Lumbar, Craniofacial, shoulder, craniosacral	HRV, ECG. BP, SC-Biopac System, Polar monitor	Immediately post to 1 year post	↑ PaSNS and SNS activity
	Jones et al. 2013 [57]	Humans- Asymptomatic and symptomatic (MS, Dementia, Cardiac issues, COPD)	STM- Foot	BP, HR, HRV, Blood flow (doppler sonography)	Immediately post to 20 min post	BP: Variable response: 2 studies demonstrating ↓ SBP only, 1 study showing ↓ SBP and DBP, 4 studies demonstrating no significant effect HR: Variable response: 3 studies demonstrating ↓ HR, 3 studies demonstrating no significant effect HRV: Significant changes in HRV in 3 studies (direction not specified) Blood Flow: Significant changes in blood flow locally Significant change in resistive index of the renal artery when kidney reflex point of foot was massaged (p < 0.001). Significant resistive index changes in the superior mesenteric artery (p = 0.021) when the intestinal reflex point on the foot was stimulated
	Xiong et al. 2015 [87]	Humans- Symptomatic (HTN)	STM- Region not specified	BP	Post treatment plan (4-16 weeks)	→ SBP vs control (MD: 3.26 (10.02, 3.49); p = 0.34) → DBP (DBP; MD: 2.41 (8.75, 3.93); p = 0.46 ↓ SBP vs antihypertensive drugs (MD: 3.47 (5.39, 1.56); p = 0.0004 → DBP vs antihypertensive drugs (MD: 0.98 (2.28, 0.32); p = 0.14)
	Lascurain-Aguirrebena et al. 2016 [61]	Humans- Symptomatic	Man- Spinal		Immediately post to 6 weeks post	↑ Sympathetic Excitation
	Schmid et al. 2008 [77]	Humans- Asymptomatic and symptomatic	Mob- Cervical	BP, SC, ST, HR, RR	Immediate post	↑ Sympathetic Excitation: ↑ SC both upper limbs ↑ HR ↑ RR ↓ DBP (During mobilization) → ST
	Chu et al. 2014 [36]	Humans- Not specified	Mob- Thoracic and cervical	SC, ST	Immediate post	
	Picchiottino et al. 2019 [69]	Humans- Asymptomatic and symptomatic	Man- Spinal Mob- Spinal, Elbow	SC, ST, Skin Blood flow, HR, HRV, RR, Pupil Diameter, Salivary α-amylase activity	During, Immediate post -10 min post	Mobilization: → ST ↑ SC compared to sham in 10/10 studies → Dermal blood flow in 1/2 studies, effects in opposite directions (both increase and decrease) in the other study → HR → BP in 2/2 studies → HRV in 1/1 study ↑ RR in 3/3 studies ↓ α-amylase activity vs sham in 1/1 study Spinal SNAG: → ST → SC Mobilization with movement: ↑ SC compared to sham in 1/1 study (elbow) ↓ or ↑ ST and skin blood flow vs sham in 1/1 study ↑ HR and ↑ BP vs sham in 1/1 study Spinal Manipulation: → HR in 3/3 studies → BP in 1 study → HRV in 7/7 studies → Pupil diameter in 1/1 study
	Sampath et al. 2024 [75]	Humans- Asymptomatic and symptomatic (HTN, Neck pain, Achilles tendinopathy)	Man- Spinal	HR, HRV, Edge light pupil cycle time, Root mean square of successive difference (rMSSD), Pupil diameter	Immediate post -5 min post	↓ in HR ↓ Low-frequency/high frequency power ratio Changes in low frequency power ↓ High frequency power ↓ Edge light pupil cycle time → Pupil diameter → HRV or BP vs sham ↑ rMSSD (HRV) vs control; →rMSSD (HRV) vs sham Changes in PR interval Statistically significant within-group Change in QRS duration
Scoping Reviews	Lima et al. 2020 [62]	Animals- (Non-Cadaveric Cats, Mice, Rabbits, Rats); healthy and induced pain	STM- Region not specified	n/a	n/a	↑ Nerve blood flow and ↓ Intraocular pressure ↓ BP ↓ HR ↑ Vascular endothelial growth factor (VEGF)-A
	Nelson 2015 [68]	Humans- Symptomatic (HTN)	STM- Region not specified	HRV, HR, Blood flow, Viscosity	n/a	↓ HRV ↓ HR in 45% of studies investigating HR * Effects do not persist beyond the MT session and are not considered clinically significant
	Rogan et al. 2023 [73]	Humans- Asymptomatic and symptomatic (Fascial pain, acute LBP, neck pain, HTN, elbow pain)	Man- Spinal Mob- Spinal STM- Paraspinal	HRV, SC, ST	Immediately post to 8 weeks post	74% demonstrated effect of single intervention on ANS ↑ SNS activity demonstrate in 51% ↑ PaSNS activity demonstrated in 24% Physiological ANS response was independent of the treatment region
Narrative Reviews	Savva et al. 2021[76]	Humans- Symptomatic	Man- Thoracic	SC, ST	n/a	↑ Sympathetic Excitation
	Field 2016 [43]	Humans- Symptomatic (Cardiac issues)	STM- Region not specified			↓ SBP and DBP
	Vicenzino 2007 [83]	Humans- Symptomatic (epicondylalgia)	Mob (MWM)- Elbow	HR, BP	n/a	↑ Sympathetic Excitation
	Yao et al. 2014 [88]	Humans- Symptomatic (Pneumonia)	Mob- Rib and thoracic	Changes is α-amylase	Immediate post	↓ α-amylase
	Potter et al. 2005 [71]	Humans- Not specified	Man- Spinal and peripheral	SC, ST, Blood flow	Immediate Post	↑ Sympathetic outflow ↑ SC ↓ ST ↓ Blood Flow
	Holey et al. 2014 [54]	Humans- Not specified	STM- Region not specified	BP, Blood Flow, ST (foot)	Immediate to two weeks post	↓ Peripheral blood flow, followed by an increase after two weeks ↑ DBP (immediate and moderate effect size) → SBP → HR → Skin Temp ↑ Sympathetic activity, with main effect was on diastolic BP rather than systolic. → Mean arterial BP ↑ ST locally at 15 min post treatment, maintained for at least 1 hour
	Weerapong et al. 2005 [86]	Humans- Asymptomatic	STM- Local and whole body	HR, BP, SC, RR	n/a	↑ vs → HR ↑ vs → BP ↑ vs → ST ↓ RR ↑ SC
	Simmonds et al. 2012 [78]	Humans- Asymptomatic	Man- Thoracic Mob- Cervical, lumbar	HRV, ST, SC	n/a	Different ANS response to noxious and non-noxious stimuli in the spine: Noxious ↑ SNS response (excitatory); non-noxious ↓ SNS response (inhibitory)
	Bolton et al. 2012 [33]	Humans- Asymptomatic	Man- Spinal Mob- Cervical	HR, ST, BP, RR, SC, Edge light pupil cycle time (ELPCT)	n/a	HR: ↓ 2 studies; ↑ 1 study; → 4 studies ST: ↓ 1 study; ↑ 1 study; → 1 study DBP: ↓ 2 studies; ↑ 1 study; → 3 studies SBP: ↓ 2 studies; ↑ 1 study; → 3 studies ↑ RR 1 study ↑ SC mobilization vs control ↑ SC HVLA vs control and vs exercise ↓ ELPCT with HVLA
	Alanez et al. 2024 [29]	Humans- Asymptomatic and symptomatic	Man- Spinal, peripheral Mob- Spinal, peripheral STM- region not specified	BP, HR, HRV, SC, ST, Pupil Diameter	n/a	→ Pupil diameter with thoracic manipulation ↓ Edge Light Pupil Cycle Time with cervical manipulation Variable response on BP (→or ↓) with all included types of MT Variable response on HR with all included types of MT Variable response on LF/HF ratio with all included types of MT

Definitions: ↑ - Increase, ↓ - Decrease, →- No change, STM- Soft tissue mobilizations, Man- Manipulation, Mob- Mobilizations, PA- Posterior to anterior, UE- Upper extremity, LBP- Low back pain, MWM- Mobilization with movement, SMT- Spinal manipulative technique, MT- Manual Therapy, ANS- Autonomic nervous system, HR- Heartrate, HRV- Heart rate variability, SC- Skin conductance, ST- Skin temperature, RR- Respiratory rate, BP- Blood pressure, SBP- Systolic blood pressure, DBP- Diastolic blood pressure, SNS- Sympathetic nervous system, PaSNS- Parasympathetic nervous system.

**Table 4:** Neurological (pain threshold) treatment mechanisms associated with MT.

	Author/Year	Subjects:	MT Technique	Measure	Time of Measure	Results/Conclusion
Systematic Reviews	Schmid et al. 2008 [77]	Humans- Asymptomatic and symptomatic	Mob- Cervical	Changes in PPT, TPT	Immediate post	↑ PPT locally and distally → TPT
	Voogt et al. 2015 [85]	Humans- Symptomatic (Musculoskeletal pain)	Man- Cervical Mob- Ankle, knee, elbow, cervical, shoulder, wrist	Changes in PPT (local) Changes in PPT (Distal)	n/a	↑ PPT In 11/14 studies (77%) * 3/11 studies the percentage of change in PPT was less than 15% ↑ PPT ipsilateral side (epicondyle) (44.2%); contralateral side (17.7%) ↓ PPT (27.3%) on the painful side (knee) and (15.3%) on remote body part
	Honoré et al. 2018 [55]	Humans- Not specified	Man- Spinal	Changes in PPT (Regional and Remote)	Immediate to 5 min post	↑ PPT vs sham in 5/8 studies ↑ PPT vs control in 2/3 studies → PPT vs HVLAT at other regions in 2/2 studies → PPT vs mobilization in 3/3 studies → PPT vs ‘other PT’ in 2/3 studies
	Coronado et al. 2013 [40]	Humans- Symptomatic (Spinal pain)	Man- Spinal	Changes in PPT	Immediate to 7 days post	→ PPT vs mobilization in 1/1 study
	Gay et al. 2013 [46]	Humans- Asymptomatic and symptomatic (Neck Pain, Scapulocostal syndrome, low back pain, breast CA survivors, Chronic tension headaches	STM- Region not specified	Changes in PPT (local and remote), Electrical pain threshold	Immediate	↑ PPT vs all other interventions (Hedges g = 0.254 [95% CI 0.105–0.403], P < 0.05) → PPT vs active treatment (Hedges g = 0.036 [95% CI -0.289–0.362], P = 0.83). ↑ PPT vs sham treatment (Hedges g = 0.268 [95% CI 0.078–0.457], P < 0.05) (I2 = 0.0%, P = 0.55) ↑ PPT vs no-treatment control (Hedges g = 0.471 [95% CI 0.113–0.830], P < 0.05)
	Jung et al. 2023 [59]	Humans- Asymptomatic and symptomatic	Man- Spinal Mob- Spinal	Changes in PPT (local)	Immediately to 15 minutes post	→ PPT (local) in patients with chronic pain: SMD = 0.25 (95 CI = -0.02 to 0.51)- low certainty evidence → PPT (local) in health controls: SMD = 0.2595 CI = -0.02 to 0.52- moderate certainty evidence
				Changes in PPT (remote)	Immediately to 15 minutes post	→ PPT remote (segmental): SMD = 0.14 (95%CI = -0.09 to 0.36) in patients with chronic pain - low certainty evidence → PPT remote (non-segmental) SMD = 0.19 (95%CI = -0.05 to 0.44) in patients with chronic pain - low certainty evidence → PPT remote (segmental): SMD = 0.19 (95% CI = -0.2 to 0.58) in healthy controls- low certainty evidence ↓ PPT remote (non-segmental): SMD = 0.26 (95%CI = 0.01 to 0.51) in healthy controls- low certainty evidence
	Coronado et al. 2012 [39]	Humans- Asymptomatic and symptomatic	Man- Spinal	Changes in PPT (Cervical, Elbow, Head, Lumbar, Trap, Web space	Immediate and delayed	↑ PPT (remote and local) vs other interventions (Hedges g = 0.315 [95% CI = 0.078; 0.552], p = 0.009) ↑ PPT (local site) (Hedges g = 0.387 [95% CI = -0.070; 0.844], p = 0.097) ↑ PPT (remote site) (Hedges g = 0.287 [95% CI = 0.073; 0.500], p = 0.008)
	Millan et al. 2012 [64]	Humans- Symptomatic (clinically induced pain)	Man- Spinal	Changes in PPT, TPT	Immediate to 7 days post	↑ PPT (19/27 studies) (4.8% to 44.2%) → TPT (6/9 studies)
	Lascurain-Aguirrebena et al. 2016 [61]	Humans- Symptomatic	Mob- Spinal		During, Immediately post to 6 weeks post	↑ PPT local and distal to site of mobilization in 5/7 studies All studies measuring thermal pain threshold reported no significant changes.
	Riley et al. 2024 [72]	Humans- Symptomatic (chronic LBP, chronic neck pain)	Man- Spinal	Changes in PPT	Immediate post to 4 weeks post	↑ PPT regionally vs other MT procedure in 1/3 studies → PPT regionally vs sham or other MT procedure in 2/3 studies
Scoping Reviews	Jun et al. 2020 [58]	Humans- Asymptomatic and symptomatic; Animals	Man- Lumbar Mob- Lumbar	Changes in PPT (local)	n/a	↑ PPT
	Lima et al. 2020 [62]	Animals- (Non-Cadaveric Cats, Mice, Rabbits, Rats); healthy and induced pain	Man- Spinal	Changes in PPT	n/a	↑ Mechanical threshold in nociceptive specific thalamic neurons → Mechanical threshold response of nociceptive specific neurons
Narrative Reviews	Vicenzino 2007 [83]	Humans- Symptomatic (epicondylalgia, ankle sprain)	Mob (MWM)- Ankle and elbow	Changes in PPT	Immediately post to post 6-sessions	→ PPT and TPT following MWM at ankle in 1 study ↓ PPT vs control for MWM at elbow (10-15%) across 3/4 studies * Magnitude of improvement in PPT was not reduced with repeated applications of over 6 successive sessions.
	Hennenhoefer et al. 2019 [52]	Humans- Not specified	Man- Region not specified	Changes in PPT, TPT	n/a	↑ PPT local and remote → TPT
	Potter et al. 2005 [71]	Humans- Not specified	Man- Spinal and peripheral	Changes in PPT	Immediate post	↑ PPT
	Savva et al. 2021 [76]	Humans- Symptomatic	Man- Spinal, elbow, wrist	Changes in PPT, TPT	n/a	↑ PPT
	Vernon 2000 [82]	Humans- Asymptomatic and symptomatic (LBP)	Man- lumbar	Changes in PPT, Electrical Pain Threshold	Immediate - 2 hrs post	↑ PPT thrust manipulation> mobilizations

Definitions: ↑ - Increase, ↓ - Decrease, → - No change, STM- Soft tissue mobilizations, Man- Manipulation, Mob- Mobilizations, PA- Posterior to anterior, UE- Upper extremity, LBP- Low back pain, MWM- Mobilization with movement, MT- Manual Therapy, HVLAT- High velocity low amplitude thrust, PT- Physical therapy, PPT- Pressure Pain Threshold, TPT- Thermal pain threshold.

**Table 5:** Neurological (other) treatment mechanisms associated with MT.

	Author/Year	Subjects:	MT Technique	Measure	Time of Measure	Results/Conclusion
Systematic Reviews Sy Sy	Tejero-Fernandez et al. 2015 [81]	Humans- Asymptomatic	STM- Region not specified	EEG	n/a	↓ Power density in β-1 in central and frontal leads
	Meyer et al. 2019 [63]	Humans- Asymptomatic and symptomatic (Spinal Pain)	Man- Spinal Mob- Spinal	Blood Oxygenation level (fMRI); EEG- Somatosensory evoked potential (SEP), Cerebellar inhibition, Mental rotation reaction-time task, Motor Evoked Potentials (MEP) Cortical Silent Period	Immediately post - 1 hr post	↑ Activation in the insular and sensorimotor cortices ↑ Activation anterior and posterior cingulate, supplementary motor area, and precentral gyrus post-SMT vs control ↓ SEP- Statistically significant pre to post (p = .02) but not vs control (p = .4) ↓ Cerebral Inhibition- post SMT vs control ↑ Motor Evoked Potential- immediately post (10 to 120 s. post-SMT vs control Regional Metabolic Rate: ↑ Broca’s area, anterior cingulate cortex, somatosensory association cortex, Wernicke’s area, visual association cortex, cerebellar vermis, and visual cortex ↓ Inferior parietal lobule, frontal pole, inferior frontal gyrus, pars triangularis, premotor area/supplementary motor area, primary motor cortex, frontal eye field, dorsolateral prefrontal cortex, angular gyrus, fusiform gyrus, inferior temporal gyrus, and temporal pole.
	Arribas-Romano et al. 2020 [31]	Humans- Symptomatic (Chronic pain)	Man- Lumbar Mob- Knee	TS, CPM	Immediately post	↑ CPM ↓ TS: The only therapeutic modality that resulted in significant change was MT for TS MT on Sensory Testing (all): Z = 1.95 (p = .05)
Scoping Reviews SC SC	Nelson 2015 [68]	Humans- Symptomatic	STM- Region not specified	EEG		↓ EEG asymmetry; Shift toward left-frontal EEG activation ↑ Blood flow to the amygdala and hypothalamus ↓ Sympathetic outflow after massage is associated with increases in structures within the brain involved in ANS regulation
	Lima et al. 2020 [62]	Animals- (Non-Cadaveric Cats, Mice, Rabbits, Rats); healthy and induced pain	Man- spinal Mob- Spinal, knee, ankle	fMRI (Spinal Cord, Cortical), Nerve Morphological Analysis	n/a	Mobilization: ↑ (9%) vs → nerve elongation ↑ Myelin sheath thickness Capsaicin injection activate areas within spinal cord dorsal horn; No statistically significant differences in activation after knee joint mobilization Capsaicin injection activate brain pain processing areas; No statistically significant differences in activation after knee joint mobilization Manipulation: ↓ Excitability (DRG neurons) STM: Non-specific changes in mean firing rate & number of short inter-spike intervals of supraoptic neuron activity → Axonal (facial nerve) branching ↓ Motor endplate poly-innervation (across 4 articles) ↑ Brachial Plexus Nerve Conduction Velocity and Nerve action potential ↑ Dendritic arborization in pyramidal cells ↑ Dendritic branching and spine density in different brain areas ↓ Neuronal activity and conduction velocity
	Jun et al. 2020 [58]	Humans- Asymptomatic and Symptomatic (low back pain); Animals	Man- Lumbar Mob- Lumbar	n/a	n/a	Four studies hypothesized changes in the CNS or sensory and motor reflex pathways to be possible mechanisms for the changes in spinal stiffness and the decrease in pain following
Narrative Review Review	Jacobson 2011 [56]	Humans- Asymptomatic	STM- Region not specified	EEG	Immediate post	Average evoked response (AER): ↑ Amplitude all levels of stimulus intensity ↓ Variability at maximum and minimum amplitudes of stimuli ↑ Capacity for efficient organization of sensory input ↑ Sensitivity to stimulation and selective inhibition as stimulus intensity increases

Definitions: ↑ - Increase, ↓ - Decrease, → - No change, STM- Soft tissue mobilizations, Man- Manipulation, Mob- Mobilizations, PA- Posterior to anterior, UE- Upper extremity, LBP- Low back pain, fMRI- Functional MRI, MWM- Mobilization with movement, SMT- Spinal manipulative technique, MT- Manual Therapy, DRG- Dorsal root ganglion, CNS- Central Nervous System, EMG- Electromyogram, ECG- Electrocardiogram, EEG- Electroencephalogram, TS- Temporal summation, MVC- Mav voluntary contraction, CPM- Conditioned pain modulation.

**Table 6:** Neurotransmitter and neuropeptide treatment mechanisms associated with MT.

	Author/Year	Subjects:	MT Technique	Measure	Time of Measure	Results/Conclusion
Systematic Reviews	Chow et al. 2021 [35]	Humans- Not specified	Man- Spinal	Plasma- Epi, NE, Substance P	5 min to 6 hrs post	→ Substance P → NE → Epi
	Sampath et al. 2017 [74]	Humans- Asymptomatic and Symptomatic	Man- Thoracic and cervical	Substance P Neurotensin Oxytocin	Immediately post	SMT vs control: immediately post ↑ Substance P (SMD-0.48, 95% CI - 0.87 to 0.10) - Low quality evidence ↑ Oxytocin (SMD -2.61,95%CI-3.5to-1.72) - Low quality evidence ↑ Neurotensin (SMD -1.8,95% CI-2.56to-1.04) - Low quality evidence → Substance P at short term follow-up (SMD -0.40, 95% CI e 1.2 to 0.4)- Very low quality evidence → Epi vs control (SMD 0.1,95%CI- 0.56to0.75) - Low quality evidence → NE vs control (SMD -0.06,95%CI-0.71to0.6)- Low quality evidence
	Araujo et al. 2019 [30]	Humans- Asymptomatic and Symptomatic (craniofacial pain, neck pain)	Man- Spinal	Plasma- Epi and NE	n/a	→ NE (low quality evidence) → Epi (low quality evidence)
	Picchiottino et al. 2019 [69]	Humans- Asymptomatic and Symptomatic	Man- Spinal Mob- Spinal, elbow	Epi and NE and NE- plasma	n/a	→ Epi → NE
	Coronado et al. 2013 [40]	Humans- Symptomatic (spinal pain)	Man- Spinal	β -endorphin	n/a	Variable results on β -endorphin; ↓ x1 study; ↑ x1 study
	Sampath et al. 2024 [75]	Humans- Asymptomatic and symptomatic (HTN, Neck pain, Achilles tendinopathy)	Man- Spinal	Plasma- Epi and NE	Immediate to 15 min post	→ NE → Epi
Scoping Review	Lima et al. 2020 [62]	Animals- (Non-Cadaveric Cats, Mice, Rabbits, Rats); healthy and induced pain	Man- Spinal Mob- Spinal, knee, ankle STM- Region not specified	n/a	n/a	Mobilization: ↓ Substance P & TRPV1 ↑ µ -opioid receptor Adenosine A1/α-2-adrenergic receptors & Serotonin mediate pain ↓ 5-HT1/2 and α2-adrenergic receptors respectively prevented and reversed joint mobilization antihyperalgesia; No statistically significant differences elicited by GABAA and opioid blockade STM: ↑ Na + , K + -ATPase Activity and NGF content
Narrative Reviews	Bolton et al. 2012 [33]	Humans- Asymptomatic	Man- Thoracic and cervical	Plasma β -endorphin	Immediate to 2 hrs post	↑ β -endorphin vs controls → β -endorphin vs sham
	Haavik et al. 2021 [49]	Humans- Not specified	Man- Spinal	Epi	n/a	→ Epi (low quality evidence from one study)
	Holey et al. 2014 [54]	Humans- Not specified	STM- Region not specified	Plasma β -endorphin	n/a	↑ β-endorphin
	Potter et al. 2005 [71]	Humans- Not specified	Man- spinal			↑ β-endorphin (x1); → β -endorphin (x1)
	Vigotsky et al. 2015 [84]	Humans- Asymptomatic and Symptomatic (Myalgia, low back pain, stage I and II Breast Cancer, Anorexia, Fibromyalgia, Autism); Animals (Rats, mice)	Man- Spinal and Peripheral Mob- Ankle, elbow STM- Region not specified	n/a	n/a	Manipulation: Inconsistent changes in β -endorphin ↑ x3; → x2 ↑ NE ↑ PEA ↑ AEA ↓ Neurotensin ↑ Orexin A ↓ Oxytocin Mobilization: ↓ Glial cell activation Soft Tissue Mobilization: Inconsistent changes in β -endorphin ↑ x1 study; → x2 studies; ↓ x1 study → β -lipotropins ↑ Oxytocin ↑ Dopamine ↑ Serotonin ↓ Arginine vasopressin ↑ Corticotropin releasing factor
	Field 2016 [43]	Humans- Not specified	STM- Region not specified	n/a		↑ Oxytocin
	Haavik et al. 2021 [49]	Humans- Not specified	Man- spinal	Neurotensin, Oxytocin, and Substance P		↑ Neurotensin vs control- Moderate quality evidence from 1 study ↑ Oxytocin vs control- Moderate quality evidence from 1 study * No difference between neurotensin and oxytocin changes vs sham HVLAT ↑ Substance P (low-quality evidence)
	Bolton et al. 2012 [33]	Humans- Asymptomatic	Man- Thoracic	Plasma Substance P	Immediate to 2 hrs post	↑ Substance P versus control

Definitions: ↑ - Increase, ↓ - Decrease, → - No change, STM- Soft tissue mobilizations, Man- Manipulation, Mob- Mobilizations, PA- Posterior to anterior, UE- Upper extremity, LBP- Low back pain, MWM- Mobilization with movement, SMT- Spinal manipulative technique, MT- Manual Therapy, NE- Norepinephrine, EPI- Epinephrine, PEA- Phenethylamine, AEA- Anandamide, HVLAT- High velocity low amplitude thrust.

**Table 7:** Neuroimmune treatment mechanisms associated with MT.

	Author/Year	Subjects:	MT Technique	Measure	Time of Measure	Results/Conclusion
Systematic Reviews	Bernier Carney et al. 2020 [32]	Humans- Symptomatic (LBP > 3 months)	STM- Gua sha	Salivary TNF-α and Heme-oxygenase (HO-1)	First session and 8^th^ session- immediate post, 30 min post	→ TNF-α → HO-1
	Chow et al. 2021 [35]	Humans- Not specified	Man- Spinal	Plasma- inflammatory markers	5 min - 12 week post	Immediate changes in the levels of selected immunological biomarkers: polymorphonuclear neutrophils, monocytes, TNF-α, IL-1β, IL-2, Ig-G, Ig-M) in asymptomatic participants vs sham manipulation or vs control groups (direction not specified) With the exception of 1 study, SMT was not associated with changes in lymphocyte levels among patients with low back pain or participants who were asymptomatic vs sham or vs control
	Sampath et al. 2017 [74]	Humans- Asymptomatic and symptomatic	Man- Thoracic and cervical	TNF-α, IL-1 PBMC, Ig-G Ig-M		Moderate quality evidence that spinal manipulation is better than control in influencing IL concentration (direction not specified)
	Tejero-Fernandez et al. 2015 [81]	Humans- Asymptomatic	STM- Region not specified	Plasma (neutrophil), Saliva (Ig-A) biopsy (cytokine profile)	n/a	↑ Neutrophils ↓ Proinflammatory cytokine production ↑ Ig-A
	Hillier et al. 2010 [53]	Humans- Symptomatic (HIV)	STM- Region not specified	Plasma CD4 cell count (cells/mm3) and viral load	n/a	↑ Natural killer cell concentration (p < 0.01) Changes in Natural Killer Cells: Z = 1.61 (p = .11); Std mean Diff.52 favoring MT ↑ CD4 cell count (p < 0.05) Changes in CD4 vs Control: Z = .96 (p = .34); Std Mean Diff:.23 favoring the control
Scoping Review	Lima et al. 2020 [62]	Animals- (Non-Cadaveric Cats, Mice, Rabbits, Rats); healthy and induced pain	Man- Spinal Mob- Spinal, knee, ankle STM- Region not specified	n/a	n/a	Mobilization: ↓ IL-1β & TNF-α in the nerve trunk & branches between treated/non-treated sides ↑ IL-10 → Leukocyte expression Manipulation: ↓ Satellitosis, c-Fos & PKCγ, IL-1β in DRG ↑ IL-10 in spinal cord STM: ↓ IL-1β ↓ Number and severity of adhesion in preventive group (i.e., massage right after surgery) ↓ Leukocyte infiltration ↓ Intraperitoneal protein and leukocyte levels → Inflammatory cell infiltration ↑ Number of thymocytes, CD4 + CD8 + , CD4 + and CD8 + ↓ TNF-α
Narrative Reviews	Colombi et al. 2019 [37]	Humans- Asymptomatic	Man- Thoracic and cervical	Venipuncture	n/a	Inconsistent changes in TNF- α and IL-2
	Haavik et al. 2021 [49]	Humans- Not specified	Man- Spinal	TNF-α and IL	n/a	Significant influence on levels of immune mediators in 18/23 studies (direction not specified) Moderate quality evidence that SMT influences IL levels (direction not specified)
	Field 2016 [43]	Humans- Symptomatic (Breast Cancer)	STM- Region not specified	n/a	n/a	↑ Natural killer cell concentration ↑ Immune Function
	Yao et al. 2014 [88]	Animals- Dogs and Rats	Mob- Rib, thoracic	White blood cell count	n/a	↑ Leukocyte mobilization and flow in dogs and rats, primarily from gut-associated lymphoid tissue ↑ White blood cells (plasma)
	Bolton et al. 2012 [33]	Humans- Asymptomatic	Man- Thoracic	TNF-α, IL1-β, Ig-G and Ig-M	Immediate to 2 hrs post	Variable changes in TNF- α (One study demonstrating ↓ and one demonstrating ↑ ) ↓ I-1β ↑ IL-2 induced Ig-G and Ig-M production
	Cook et al. 2024 [38]	Humans and animals- not specified	Man- Spinal and Peripheral Mob- Spinal and Peripheral STM- Region not specified	Pro-inflammatory and anti-inflammatory cytokine profiles	n/a	↑ IL-2 ↓ IL-4 → IL-8, IL-12, IL-17, IL-23, GM-CSF Changes (variable direction) in IL-13, IL-10, TNF-α, IFN-y, IL-1 β, IL-6 Changes in TNF-α in the short term; vary depending on the technique selected and the comorbidities of the patient

Definitions: ↑ - Increase, ↓ - Decrease, → - No change, STM- Soft tissue mobilizations, Man- Manipulation, Mob- Mobilizations, PA- Posterior to anterior, UE- Upper extremity, LBP- Low back pain, MCP- Metacarpophalangeal, MWM- Mobilization with movement, SMT- Spinal manipulative technique, MT- Manual Therapy, TNF- Tumor Necrosis Factor, IL- Interleukin, PBMC- Peripheral blood mononuclear cells, Ig- Immunoglobin, DRG- Dorsal root ganglion, PKC- Protein kinase C.

**Table 8:** Neuroendocrine treatment mechanisms associated with MT.

	Author/Year	Subjects:	MT Technique	Measure	Time of Measure	Results/Conclusion
Systematic Reviews	Bernier Carney et al. 2020 [32]	Humans- Parkinson’s Disease	STM- Region not specified	Salivary cortisol (concentration and total secretion)	First session and 8^th^ session- immediate post, 30 min post	First session: Immediately post: ↓ Cortisol MT group (p = .002) 30 min post: ↓ Cortisol both MT (p = .0003) and control (p = .016) Eighth session: Immediately post: ↓ Cortisol MT (p = .003) and control (p = .028) 30 min post: ↓ Cortisol MT (p = .0006) and control (p = .027) Total cortisol secretion: ↓ immediately after the eighth intervention in the MT (p = .003) and control (p = .035) groups; remained ↓ in MT group (p = .004) 30-min post intervention
	Chow et al. 2021 [35]	Humans- Asymptomatic and symptomatic	Man- Spinal	Plasma- Testosterone, T/C Ratio, O2Hb, salivary cortisol	5min - 6 hrs post	→ Testosterone → T/C ratio → Oxyhemoglobin Changes in the level of salivary cortisol in the immediate term (direction not specified) among asymptomatic participants compared with sham SMT
	Sampath et al. 2017 [74]	Humans- Asymptomatic and symptomatic	Man- Thoracic and Cervical	Plasma cortisol	5min - 2hrs post	SMT> control (SMD -0.46, 95% CI - 0.93 to 0) in reducing cortisol levels immediately after intervention. (low quality evidence)
	Coronado et al. 2013 [40]	Humans- Symptomatic (Spinal Pain)	Man- Spinal	Cortisol	n/a	↓ Cortisol in 1/1 study
	Tejero-Fernandez et al. 2015 [81]	Humans- Asymptomatic	Man- Region not specified	Salivary and plasma cortisol	n/a	↑ Cortisol massage and control group without significant difference
	Sampath et al. 2024 [75]	Humans- Asymptomatic and symptomatic (HTN, Neck pain, Achilles tendinopathy)	Man- Spinal	Plasma Cortisol, Testosterone/Cortisol ratio	Immediate to 6 hours post	Significant between-group difference of salivary cortisol 5 min post (0.35, p < 0.01) Significant between-group difference T/C ratio 6 hr post (-0.09, p < 0.01) in the intervention group
Narrative Reviews	Field 2016 [43]	Humans- Symptomatic (Cardiac issues)	STM- Region not specified	n/a	n/a	↓ Cortisol
	Colombi et al. 2019 [37]	Humans- Asymptomatic and symptomatic (neck pain)	Man- Thoracic and Cervical	Saliva (n = 5); Venipuncture (n = 4)	Immediate to 2 hrs post	→ Cortisol in symptomatic patients (3/3 studies)
	Haavik et al. 2021 [49]	Humans- Not specified	Man- Spinal	Cortisol		↑ Cortisol
	Moyer et al. 2011 [66]	Humans- Asymptomatic and symptomatic	STM- Full Body, Neck and Shoulders, Feet, Back, Upper body	Blood, urinary, and salivary cortisol	Immediate to 6 weeks post	→ Cortisol: Single session (first)- Massage versus control (d = 0.15, 95% CI = 0.04, 0.34) → Cortisol: Single session (last)- Massage versus control (d = 0.15, 95% CI = 0.08, 0.37) → Cortisol: Multiple doses- Massage versus control (d = 0.12, 95% CI = 0.05, 0.28) Within Group: ↓ Cortisol range 10.8% (single-dose reduction at first treatment) to 35.0%
	Nelson 2015 [68]	Humans- Symptomatic	STM- Region not specified	Cortisol	n/a	→ Cortisol (effect is very small or not statistically distinguishable from zero)
	Bolton et al. 2012 [33]	Humans- Asymptomatic	Man- Thoracic and cervical	Plasma Cortisol	Immediate to 2 hrs post	→ Cortisol vs control or sham

Definitions: ↑ - Increase, ↓ - Decrease, → - No change, STM- Soft tissue mobilizations, Man- Manipulation, Mob- Mobilizations, SMT- Spinal manipulative technique, MT- Manual Therapy.

**Table 9:** Neuromuscular treatment mechanisms associated with MT.

	Author/Year	Subjects:	MT Technique	Measure	Time of Measure	Results/Conclusion
Systematic Reviews	Corso et al. 2019 [41]	Humans- Asymptomatic	Man- Spinal	MVC per surface EMG, Transverse Abdominus (TA) Ultrasound	Immediate post to 60 min post	↑ MVC vs control with PROM immediately post in plantar flexors and quad; 30 minute post in plantar flexors only; no difference at 60 min post in either group → Quad MVC after L3–4 SMT vs sham manipulation → TA thickness measured with multiple ultrasound readings at rest or during contraction vs sham
	Schmid et al. 2008 [77]	Humans- Asymptomatic and Symptomatic	Mob- Cervical	EMG	Immediate post	↓EMG activity of the superficial neck flexor in symptomatic patients → EMG activity in healthy controls
Scoping Reviews	Lima et al. 2020 [62]	Animals- (Non-Cadaveric Cats, Mice, Rabbits, Rats); healthy and induced pain	Man- Spinal Mob- Spinal, knee, ankle	n/a	n/a	Mobilization: → Muscle spindle discharge upon chemosensitization of small diameter afferents Manipulation: Trunk GTO & muscle spindle afferent discharge to impulse phase of SM ↑ Afferent discharge at 100ms ↑ Primary & secondary muscle spindle discharge over time profile ↑ EMG response with Variations in force ↑ EMG as force ↑ ; No statistically significant differences in EMG for pulse duration ↑ Compound action potentials for shorter pulse durations ↓ (25-30%) positive EMG response in the degenerative group; no statistically significant differences in spondylosis group. ↑ Resting muscle spindle discharge at 2mm either increased or decreased at 3mm ↑ Muscle spindle discharge during manipulation: ↑ position discharge & ↓ resting discharge upon longer & higher magnitude preload ↑ Spindle discharge at all contact sites with higher responsiveness at the targeted vertebra ↑ Spindle discharge with Lower thrust durations (<150ms) ↓ Spindle discharge &> 10s to return to baseline activity ↓ Spontaneous neuronal activity; attenuation of inhibitory evoked response on the contralateral hind-paw
	Jun et al. 2020 [58]	Humans- Asymptomatic and Symptomatic (low back pain); Animals	Man- Lumbar Mob- Spinal	LM Recruitment	n/a	Six studies suggested a link between a change in muscle activity or recruitment and the change in spinal stiffness: change in LM thickness ratio, ↑ LM recruitment, ↓ muscle activity of the erector spinae, facilitation of muscle activity, presence of muscle relaxation, correction of an altered motor function, and ↓ involuntary muscle activity
Narrative Reviews	Potter et al. 2005 [71]	Humans- Not specified	Man- Spinal and peripheral	EMG reflex	During and immediately post	Reflex response elicited with latency around 4 ms. ↓ EMG activity Changes in α motor neuron activity (H-reflex) - either ↑ (2 studies) or ↓ (2 studies) ↑ Post-treatment MVC as compared to a sham manipulation and control
	Jacobson 2011 [56]	Humans- Asymptomatic	STM- Region not specified	EMG	Immediate post	↑ Rhythmic coherence ↑ Functional independence of muscle activation ↑ Amplitude of muscle contraction ↓ Agonist–antagonist co-contraction activation more specific to locus of primary action ↑ Differentiation between isometric and isotonic contractions movements smoother, larger, and less constrained, fewer extraneous movements; spatial excursions more dynamic and energetic ↑ Activation of stabilizing muscles during tasks ↓ Estimated energy expenditure
	Haavik et al. 2021 (2) [48]	Humans- Not specified	Man- spinal	EMG, H-Reflex	n/a	Changes in neuromuscular function that occur after spinal adjustments impact the CNS and are primarily due to supraspinal excitability changes, and to a lesser degree, due to spinal cord excitability changes SMT alters supraspinal excitability SMT activates the deep muscle afferents from paravertebral tissues, particularly activating the muscle spindles and potentially Golgi tendon organs
	Hennenhoefer et al. 2019 [52]	Humans- Not specified	Man- Region not specified	Muscle Tone- EMG, H-Reflex	n/a	Stabilization of motor neuron pool to correct for aberrant postural and proprioceptive behavior
	Bolton et al. 2012 [33]	Humans- Asymptomatic	Man- Sacral	Phasic perineal contractions (PPC)	n/a	↑ PPC
	Pickar 2002 [70]	Humans- Asymptomatic and Symptomatic	Man- Spinal	EMG		↓ or ↑ Paraspinal muscle activity

Definitions: ↑ - Increase, ↓- Decrease, → - No change, STM- Soft tissue mobilizations, Man- Manipulation, Mob- Mobilizations, PA- Posterior to anterior, UE- Upper extremity, LBP- Low back pain, LM- Lumbar Multifidi, MCP- Metacarpophalangeal, MWM- Mobilization with movement, SMT- Spinal manipulative technique, MT- Manual Therapy, GTO- Golgi tendon organ, EMG- Electromyogram, MVC- Mav voluntary contraction.

**Table 10:** Other mechanisms associated with MT.

	Author/Year	Subjects:	MT Technique	Measure	Time of Measure	Results/Conclusion
Systematic Reviews	Tejero-Fernandez et al. 2015 [81]	Humans- Asymptomatic	STM- Region not specified	Plasma Creatine kinase measures	n/a	↓ CK ↑ Mitochondrial biogenesis
	Corso et al. 2019 [41]	Humans- Asymptomatic	Man- spinal	MIP, MEP, TLC, residual volume Vo2 Max Blood Lactate Concentration ECG	Immediate post	→ Rate of perceived exertion (RPE) (treadmill or ergometer) → VO2 max → Blood lactate concentration → MIP → MEP → TLC
Scoping Reviews	Nelson 2015 [68]	Humans- Symptomatic	STM- Region not specified	n/a	n/a	↑ O2 saturation ↓ Central venous pressure ↓ Pulmonary vessel resistance
	Lima et al. 2020 [62]	Animals- (Non-Cadaveric Cats, Mice, Rabbits, Rats); healthy and induced pain	Man- Spinal Mob- Spinal, knee, ankle STM- Region not specified	n/a	n/a	Mobilization: Prevention of Oxidative stress & increase in protein carbonyls and catalase → Mitochondrial function & superoxide dismutase ↑ Collapsin response mediator protein-2, galactokinase 1, tropomyosin-4, and transthyretin expressions → Gene expression observed with mobilization Pain ↓ is mediated by Cannabinoid subtype receptor 1 and 2 availability Opioid receptors mediate pain ↓ ↓ CD11b/c and GFAP glycoproteins Manipulation: ↓ Excitability Satellitosis (glial cells) Prevention of ↑ in lipid hydroperoxides and NO metabolites; ↓ of catalase activity STM: ↑ Transport of subcutaneous nanoparticles to lymph node. ↑ Liposome transport into bloodstream ↓ iNOS, COX-2, NO, PGE2, MMP-1, and proteoglycan syntheses ↓ Insulin, gastrin & somatostatin; and ↑ glucose ↓ Gene Expression: ↓TAK1 to inhibit NF-ĸB activation ↓ Matrix metalloprotease-13 & Collagen II; No differences in Collagen ↑ Gene expression in the liver ↓ Differential gene expression ↓ Intraperitoneal protein ↓ Noradrenergic innervation of lymphoid organs ↓ Tissue-type plasminogen activator and plasminogen activator inhibitor-1 levels ↑ Intestinal function: ↑ c-kit mRNA and protein levels ↑ Number, distribution, and ultrastructure of colonic interstitial cells ↓ Cohesive adhesion; delayed M2 migration intraperitoneally. ↑ Protein levels & DNA synthesis. ↑ FGF-2 and CD34 proteins Activation of MrgprB4 + neurons upon brushing stimulus
Narrative Reviews	Field 2016 [43]	Humans- Not specified	STM- Region not specified	n/a		↓ Bilirubin levels
	Jacobson 2011 [56]	Humans- Asymptomatic	STM- Region not specified	Plasma	Immediate post	↑ Serum glutamic ↑ Oxalacetic transaminase
	Bolton et al. 2012 [33]	Humans- Asymptomatic	Man- Spinal Mob- Spinal	FVC, FEV-1, Freq. and amp. gastric contractions – electrogastrogram Plasma ACTH	n/a	↑ FVC and FEV-1 ↑ FVC and FEV-1 with HVLA vs non-thrust ↓ Frequency and ↑ amplitude of gastric contractions post Cervical SMT → ACTH vs sham

Definitions: ↑ - Increase, ↓ - Decrease, → - No change, STM- Soft tissue mobilizations, Man- Manipulation, Mob- Mobilizations, PA- Posterior to anterior, UE- Upper extremity, LBP- Low back pain, MCP- Metacarpophalangeal, MWM- Mobilization with movement, SMT- Spinal manipulative technique, MT- Manual Therapy, MIP- Max inspiratory pressure, MEP- Max expiratory pressure, TLC- Total lung capacity, FVC- Forced vital capacity, FEV-1- Forced expiratory volume in 1 seconds.

**Table 11:** Treatment mechanisms by domain and level of literature support.

Domains and mechanisms		Supported by:				
		Systematic Review(s)			Scoping Review(s)	Narrative Review(s)
		Moderate Quality	Low Quality	Critically low Quality		
Biomechanical	↓ Scar tissue					[43,50]
	Joint movement during (lumbar)			[61]		[42]
	Gapping of zygapophyseal joints					[42]
	↑ Neuroforaminal space			[89]		[71]
	↑ Disc Height					[45]
	Joint position post (Metacarpophalangeal)					[70,71,83]
	Joint position post (spine)			[33]		[70]
	↓ Disc protrusion					[45]
	↑ Intradiscal pressure				[58]	[45]
	Changes in disc physiology^+^			[65,89]	[58]	[45]
	↓ Myofibril damage				[62]	
	No change in muscle volume				[62]	
	↑ Cross sectional area				[62]	
	Change in viscoelastic properties of soft tissues^+^			[89]	[58]	
Neurovascular	Sympathetic excitation		[60]	[34,61,77]	[73]	[54,71,76,78,83]
	Parasympathetic excitation			[34]	[73]	
	↑ Skin conductance	[30]	[60]	[36,51,67,69,77,90]		[33,71,86]
	↑ Respiratory rate		[60]	[69,77]		[33]
	↑ Blood pressure		[60]			[86]
	↑ Heart rate		[60]			
	↓ Skin temperature	[30]	[60]	[36,67,90]		[71]
	Changes in Heart rate variability^+^		[57,75]		[68]	
	Blood pressure changes^+^		[57]	[44,51,77,80]		[43]
	↓ α -amylase			[51,69]		[88]
	↑ Vascular endothelial growth factor -A				[62]	
Neurological	↑ Pressure pain threshold (local)	[46,55,72]	[64]	[39,77,85]	[58]	[52,71,76,82]
	↑ Pressure pain threshold (remote)	[46,55]		[39,77,85]		[52]
	Functional MRI (fMRI) Changes^+^		[63]			
	Electroencephalogram (EEG) Changes^+^		[63]	[81]	[62,68]	[56]
	↓ Temporal summation		[31]			
	↑ Conditioned pain modulation		[31]			
Neurotransmitter & Neuropeptide	No change in Epinephrine	[30]	[35,74,75]	[69]		[49]
	No change in Norepinephrine	[30]	[35,74,75]	[69]		
	Changes in Substance P^+^		[74]		[62]	[33,49]
	Changes in Neurotensin^+^		[74]			[49,84]
	Changes in β-endorphin^+^			[40]		[33,54,71,84]
	Changes in Oxytocin (direction not specified)^+^		[74]			[43,49,84]
	↑ Phenethylamine					[84]
	↑ Anandamide					[84]
	↑ Orexin A					[84]
	↑ Dopamine					[84]
	↑ Serotonin					[84]
	↑ Corticotropin releasing factor					[84]
	↑ Na+, K+ -ATPase Activity and NGF content				[62]	
Neuroimmune	↑ Neutrophils			[81]		
	↓ Proinflammatory cytokines			[81]	[62]	[38]
	↑ Immunoglobin A			[81]		
	↑ Natural killer cell concentration		[53]			[43,38]
	↑ CD4 cell count		[53]		[62]	
	↓ Anti-inflammatory cytokines				[62]	[38]
	Changes in immune markers^+^		[35,74]	[81]	[62]	[33,37,38,43,49]
	↑ White blood cells					[88]
Neuro-endocrine	↓ Cortisol		[74]	[32,40]		[43]
	Changes in cortisol^+^		[35,75]			[49]
	Changes in Testosterone/Cortisol ratio		[75]			
Neuromuscular	↑ Maximal voluntary contraction		[41]			[71]
	↓ EMG activity in compensatory musculature			[77]		[52,56,71]
	↓ Spontaneous neuronal activity				[62]	
	↓ Involuntary muscle activity				[58]	
	↑ Amplitude of muscle contraction					[56]
	↓ Agonist–antagonist co-contraction					[56]
	↑ Activation of stabilizing muscles during tasks					[56]
	Changes in paraspinal muscle activity^+^					[70]
Other	↓ Creatine Kinase			[81]		
	↑ Mitochondrial biogenesis			[81]		
	↑ O^2^ saturation				[68]	
	↓ Central venous pressure				[68]	
	↓ Pulmonary vessel resistance				[68]	
	↓ CD11b/c and GFAP glycoproteins				[62]	
	↓ Excitability Satellitosis (glial cells)				[62]	
	↑ Transport subcutaneous nanoparticles to lymph node				[62]	
	↑ Liposome transport into bloodstream				[62]	
	↓ Gene Expression				[62]	
	↑ Intestinal function				[62]	
	↑ Serum glutamic					[56]
	↑ Forced vital capacity					[33]
	↑ Forced expiratory volume capacity					[33]

+ =  Changes variable direction or not specified

### Quality assessment and risk of bias

Thirty-nine reviews were appropriate for quality and risk of bias appraisal. The included reviews were primarily of critically low (n = 23) to low-quality (n = 12) with the exception of four reviews of moderate quality. Overall quality of the included reviews is presented in Table 12 with itemized scoring presented in S5 Appendix. Fourteen reviews were rated at high risk of bias and twenty-five rated as low risk of bias. Overall risk of bias of the included reviews is presented in Table 12 with itemized scoring presented in S6 Appendix.

**Table 12:** Risk of bias and quality of included systematic reviews.

Author(s)	Risk of Bias (ROBIS)	Quality (AMSTAR-2)
Araujo et al. 2019 [30]	Low	Moderate
Arribas-Romano et al. 2020 [31]	Low	Low
Bernier Carney et al. 2020 [32]	Low	Critically Low
Borges et al 2018 [34]	High	Critically Low
Chow et al. 2021 [35]	Low	Low
Chu et al. 2014 [36]	Low	Critically Low
Coronado et al. 2013 [40]	Low	Critically Low
Coronado et al. 2012 [39]	Low	Critically Low
Corso et al. 2019 [41]	Low	Low
Galindez-Ibarbengoetxea et al. 2017 [44]	High	Critically Low
Gay et al. 2013 [46]	Low	Moderate
Gera et al. 2020 [47]	High	Low
Hegedus et al. 2011 [51]	Low	Critically Low
Hillier et al. 2010 [53]	Low	Low
Honoré et al. 2018 [55]	High	Moderate
Jones et al. 2013 [57]	High	Low
Jun et al. 2020 [58]	Low	Critically Low
Jung et al. 2023 [59]	Low	Low
Kingston et al. 2014 [60]	Low	Low
Lascurain-Aguirrebeña et al. 2016 [61]	High	Critically Low
Lima et al. 2020 [62]	High	Critically Low
Meyer et al.2019 [63]	Low	Low
Millan et al. 2012 [64]	Low	Low
Mitchell et al. 2017 [65]	Low	Critically Low
Navarro-Santana et al. 2020 [67]	Low	Critically Low
Nelson 2015 [68]	High	Critically Low
Picchiottino et al. 2019 [69]	Low	Critically Low
Riley et al. 2024 [72]	Low	Moderate
Rogan et al. 2023 [73]	High	Critically Low
Sampath et al. 2017 [74]	Low	Low
Sampath et al. 2024 [75]	Low	Low
Schmid et al. 2008 [77]	Low	Critically Low
Sousa et al. 2020 [79]	High	Critically Low
Sullivan et al. 2020 [80]	High	Critically Low
Tejero-Fernandez et al. 2015 [81]	High	Critically Low
Voogt et al. 2015 [85]	Low	Critically Low
Xiong et al. 2015 [87]	High	Critically Low
Young et al. 2024 [33]	Low	Critically Low
Zegarra-Parodi et al. 2015 [90]	High	Critically Low

### Biomechanical mechanisms

Fourteen reviews of critically low quality reported biomechanical treatment mechanisms associated with MT. (Table 2) Five reviews reported changes in joint position associated with MT techniques [42,61,70,83,89]. One of these reviews reported no correlation between joint changes and improvement in pain or impairment [83]. Two reviews questioned the concept of joint position changes with MT, most specifically at the cervical spine [52,61]. Five reviews supported physiological changes in soft tissue associated with MT (such as viscoelastic properties) [43,50,58,62,89]. Four reviews reported changes in disc characteristics following MT techniques (e.g., intradiscal pressure) [45,58,65,89]. All four reviews supported increased disc diffusion with two of these studies supporting translational association with improved clinical outcomes [45,65].

### Neurovascular mechanisms

Thirty-two studies of critically low to moderate quality reported neurovascular mechanisms associated with MT. (Table 3) Twelve of the included reviews favored sympathoexcitation across outcome measures [30,34,36,54,60,61,71,73,76,77,83,86]. One review favored sympathoexcitation if the MT technique was noxious and sympathoinhibition if the technique was non-noxious [78]. A decrease in alpha-amylase levels, a proposed measure of Autonomic Nervous System (ANS) function, was reported across 3/3 reviews, indicating a sympathoinhibitory effect of MT [51,69,88]. Increased skin conductance was reported in 12 reviews following MT intervention [30,33,36,51,60,67,69,71,73,77,86,90]. No change in skin temperature was reported in 14 reviews post MT intervention [30,33,36,51,54,60,67,69,71,73,77,78,86,90]. One review, however, reported inverse responses related to both skin conductance and skin temperature in individuals with LBP [90] while other symptomatic populations did not demonstrate the same effect. Heart rate, heart rate variability and blood pressure demonstrated a change of variable direction without a clear rationale for variations.

### Neurological mechanisms

Twenty-three reviews of critically low to moderate quality reported neurological treatment mechanisms associated with MT. Twenty reviews investigated changes in pain threshold following MT application (Table 4). Increases in local pressure pain threshold (PPT) versus control and sham were demonstrated in 12 reviews [39,46,55,58,61,62,64,71,76,77,82,85]. Two reported no difference in effect between mobilization and manipulation [40,55],one reported larger PPT increase in the manipulation group [82] and one reported mixed results [72]. Two reviews reported no difference in PPT between MT and active PT management [46,55]. Several reviews reported the remote effect of MT on PPT with general support for an increase in PPT however not consistent across reviews. Four reviews reported no effect of MT on thermal pain threshold (TPT) [64,77,83]. Seven reviews reported other neurological mechanisms (Table 5), including changes in EEG activity [56,63,68,81], nerve characteristics [62], and cerebral blood flow [62,63]. Improved conditioned pain modulation (CPM) and reduced temporal summation (TS) were supported by 1 review [31].

### Neurotransmitter/neuropeptide mechanisms

Sixteen reviews of critically low to moderate quality reported neurotransmitter and/or neuropeptide treatment mechanisms associated with MT. (Table 6) Increase in oxytocin levels post MT application was reported in 4 reviews [43,49,74,84] with the exception of 1 review reporting increased levels with STM and decreased levels with manipulation [84]. Substance P was included in 5 reviews with 3 reviews on spinal manipulation favoring an increase, 1 review on mobilizations favoring a decrease, and 1 review on spinal manipulation favoring no change [33,35,49,62,74]. Increased β-endorphin was reported in 5 reviews following MT application versus control, however less consistent results and less significant changes when compared with sham intervention [33,40,54,71,84]. Little to no change in Norepinephrine (NE) and Epinephrine (Epi) levels with MT were reported in 7 reviews based on low quality evidence [30,35,49,69,74,75,84].

### Neuroimmune mechanisms

Twelve reviews of critically low to low quality reported neuroimmune treatment mechanisms associated with MT. (Table 7) General support was demonstrated for changes in cytokine levels with MT application. Trends towards a decrease in pro-inflammatory cytokines (IL-1β, TNF-α) and an increase in anti-inflammatory cytokines (IL-2, IL-10) were seen across reviews with some variability. This was supported across symptomatic and asymptomatic populations and was more significant with MT application than control and sham interventions. Other immune markers including leukocytes [62,81,88], natural killer cells [43,53], Immunoglobin (Ig)-A [81], Ig-G [33,35], and Ig-M [33,35] also demonstrated modulation with MT intervention.

### Neuroendocrine mechanisms

Twelve reviews of critically low to low quality reported neuroendocrine treatment mechanisms associated with MT. (Table 8) All reviews investigated changes in cortisol levels with general support for modulation of variable direction and effect sizes. Little to no difference from sham and control was reported in 5 reviews [32,33,66,68,81], larger response in MT groups reported in two reviews [35,74], and longer carryover of effects with MT vs control was reported in one review [32].

### Neuromuscular mechanisms

Ten reviews of critically low to low quality reported neuromuscular mechanisms associated with MT. (Table 9) Lima et al. assessed muscle activity during mobilization and manipulation and reported changes in muscle spindle afferent discharge, which demonstrated variability based on targeted segment and thrust velocity [62]. Post treatment responses across included reviews support increased maximum voluntary contraction [41,56,71], reduced EMG activity [62,70,71,77], and reduced muscle interference during contraction [52,56,58,70].

### Other mechanisms

Six reviews of critically low quality included treatment mechanisms outside of the previous domains. (Table 10) Three studies investigated cardiopulmonary responses to MT. Results suggested increased forced vital capacity, forced expiratory volume, and O_2_ saturation without supporting changes in VO_2_ Max, total lung capacity, blood lactate levels, or other reported measures of cardiopulmonary function. [33,41,68]. Other reviews reported on changes in gene expression [62], intestinal function [62], mitochondrial function [62,81] and enzyme, protein and amino acid profiles [56,62,81].

## Discussion

Findings from this review support complex multisystem biomechanical, neurovascular, neurological, neurotransmitter/neuropeptide, neuroimmune, neuroendocrine, neuromuscular, and other mechanistic responses occurring with the application of MT. The overall quality of evidence supporting these responses was critically low to moderate, therefore these results should be interpreted with caution. Furthermore, care should be taken in assuming translation to clinical relevance as these processes are influenced by a multitude of intrinsic and extrinsic factors and are likely to demonstrate variability between individuals. Some included reviews attempted to establish clinical relevance, including Jun et al. [58], which reported correlation between being a positive responder to spinal manipulation, and improved multifidi recruitment post technique application; however, non-investigated translation to clinical outcomes should not be assumed. For example, neuroimmune treatment mechanisms favor an increase in anti-inflammatory mediators and decrease in inflammatory mediators. These changes are not unique to MT [38] and the relevance of these changes to immune system status was questioned in several of the included reviews [35,49] and furthermore by the chiropractic community in a recent statement paper [91].

Overall, the current review supports peripheral, segmental spinal, and supraspinal neurological mechanisms occurring with the application of MT, which can be measured directly or indirectly. MT has been shown to attenuate nociceptive spinal excitability [92]. This alteration in neural excitability as well as changes in tissue sensitivity as measured via PPT are theorized to occur due to facilitation of descending inhibitory mechanisms [93]. Previous animal model research [94] demonstrated that joint-based MT likely induces analgesia via non-opioidergic inhibitory pathways, that include noradrenergic and serotonergic mechanisms in the central nervous system. Changes in tissue sensitivity may also be related to changes in functional connectivity involving the PAG [95]. One included review [46] reported that 11 studies reported correlation between hypoalgesia and clinical pain reduction; however, the degree to which changes in quantitative sensory testing translate to clinical outcomes has demonstrated question [96–98], likely due to poor baseline assessment of which aberrant neurophysiological mechanisms are present within the study participant group. Descending regulatory mechanisms have also been proposed to influence neuroimmune [99,100], neuroendocrine [101], neurovascular [101], and neuromuscular responses [102]. The crosstalk between mechanisms further complicates research within this field, as discussed in a recent review on neuroimmune mechanisms associated with MT [38].

While historic models of MT promote assessment and treatment based on biomechanical principles, the overwhelming majority of mechanisms studied suggest stronger support for neurological changes than biomechanical. These findings agree with several recent reviews suggesting non-specific analgesic effects associated with MT [103,104]. Several recent publications have emphasized the need for updated training paradigms and modernized practice patterns to accommodate what is currently known and unknown regarding these complex mechanistic responses [12,105–108]. This review summarizes treatment mechanisms associated with MT application supporting complex multisystem responses; however, care must be taken in interpreting these findings due to two distinct realizations:

1) Treatment mechanisms that occur are unlikely to be unique to a specific MT intervention. It is unclear based on the results of this review which mechanisms are specific to MT versus those which are related to the fact that ‘an intervention’ was applied. This can be seen with the significant reduction in effect size across several treatment mechanisms when compared to other active controls and sham techniques. Recent work has cited the importance of better understanding the specific and shared mechanisms associated with MT [109].
2) Treatment mechanisms that occur are unlikely to be consistent across different populations. Contextual factors such as patient factors, provider factors, and environmental factors have been shown to influence mechanistic response to MT forces and therefore should be considered when investigating and interpreting mechanistic responses to MT. While contextual factors have been solidified as a critical component of mechanistic response, two recent studies obtained consensus on gaps within this field specifically related to MT treatment mechanisms [110,111].

### Clinical implications

Clinicians using MT interventions should appreciate that multisystem mechanisms occur beyond the local tissue to which MT is targeted. While not reasonable to assess these mechanisms clinically in real time, understanding the complex interactions across systems regulating processes such as immune function, cardiovascular function, and neuroendocrine function are important factors to consider. Many of the included reviews looked at these mechanistic responses in isolation and clinicians should appreciate the complex interaction between these systems producing the observed response which they see clinically are the sum of these mechanisms with interaction from contextual variables.

### Research implications

High quality primary and secondary research within this area of study is needed to further investigate changes in the mechanistic domains outlined within this review. Researchers should address identified gaps within MT mechanisms research [110,111]. Collaboration between clinical researchers and basic scientists should be leveraged to establish translational value of these mechanisms to clinical outcomes, assess contextual factor influence on both mechanistic and clinical response, and determine if there are pain phenotypes which provide different mechanistic and clinical responses. Research should be focused on symptomatic patient populations to promote relevance of findings. Furthermore, emphasis should be placed on study designs effective in establishing causation such as those which have been proposed in previous work [6].

### Limitations

Several limitations are present which must be considered in interpreting the findings of this review. Although reporting of different MT techniques occurred, this review did not attempt to differentiate between treatment mechanisms for different MT techniques (STM, manipulation, mobilization). Several of the included reviews did report on these findings in their results. Significant heterogeneity was present between the included reviews related to type of MT technique, type of mechanistic measure, and subjects. Several of the included reviews were not in English which required translation, therefore language barriers may have been a limitation during screening, quality assessment, risk of bias assessment, and data extraction. Furthermore, this review included narrative reviews which are less structured and transparent than systematic/scoping reviews allowing increased potential for bias. These reviews were included to broaden the investigated mechanisms and the results demonstrated beneficial information extracted from these reviews which would have otherwise been excluded. Inclusion criteria were used (search strategy outlined in review) in an attempt to include more transparent narrative reviews.

### Summary

This review supports multisystem mechanistic responses with the application of hands-on intervention across the biomechanical, neurological, neurovascular, neuroendocrine, neurotransmitter/neuropeptide, and neuromuscular domains. The clinical value of these mechanistic responses has not been well established. Clinicians should appreciate the uncertainty related to ‘why’ these interventions work and future research should look to better define which mechanisms (or combinations of mechanisms) are mediators of clinical response.

### Statement on living review

With permission from the publisher, results from this living review will be housed at the Duke Center for Excellence in Manual and Manipulative Therapy (CEMMT) website at https://sites.duke.edu/cemmt/. Results will be updated every 6 months to include newly published research. Please reach out to the corresponding author with any potential reviews which may meet the inclusion criteria to be included on the next revision.

## Supporting information

S1 Appendix Definition of mechanisms table. (PDF)

S2 Appendix Search strategy. (PDF)

S3 Appendix Excluded Full texts. (PDF)

S4 Appendix Extracted data. (PDF)

S5 Appendix Quality scores (AMSTAR-2). (PDF)

S6 Appendix Risk of bias scores (ROBIS). (PDF)

S1 Checklist PRISMA 2020 Checklist. (DOCX)
