# Supplementary material for: The mechanisms of manual therapy: A living review of systematic, narrative, and scoping reviews
Source: PLoS One. 2025 Mar 18;20(3):e0319586. doi: 10.1371/journal.pone.0319586 (PMC11918397; doi:10.1371/journal.pone.0319586)
Supplement: S1 Appendix — (PDF) [file pone.0319586.s001.pdf]

Appendix A: Defined mechanistic domains and examples of direct and indirect measures of these domains.

Mechanistic Domain	Definition	Examples	Direct Measure	Indirect Measure
Biomechanical	Measures of molecular, cellular or physiological processes representative of changes in the position, structure, fluid dynamics or movement characteristics of tissue.	Joint position changes, tissue movement, fluid loading	Imaging	
Neurological	Measures of molecular, cellular or physiological processes representative of changes in central nervous system and/or peripheral nervous system activity.	Changes in neural conduction	MEG, EEG, NCV	fMRI, conditioned pain modulation, temporal summation, pressure pain threshold
Neuroimmune	Measures of molecular, cellular or physiological processes representative of changes in neuroimmune system status.	Inflammatory and anti-inflammatory mediator changes	MEG, EEG (subcortical)	Inflammatory cytokine, anti-inflammatory cytokine, Leukocyte, Lymphocyte, Neutrophil, and Immunoglobin profile. Vagus nerve activation
Neurovascular	Measures of molecular, cellular or physiological processes representative of changes in blood flow centrally or peripherally.	ANS response, blood flow changes locally and remotely	Echocardiogram, Vascular ultrasound	Skin conductance, skin temperature, heart rate, heart rate variability, blood pressure, Alpha-amylase concentration, dermal blood flow
Neurotransmitter and neuropeptide	Measures of molecular, cellular or physiological processes representative of changes in cellular messengers	Changes in neurotransmitter and neuropeptide levels (Plasma, Saliva)		Norepinephrine, Epinephrine, Serotonin, Phenethylamine, Anandamide, Neurotensin, Oxytocin, Substance P, Beta-Endorphin, Orexin A
Neuroendocrine	Measures of molecular, cellular or physiological processes representative of changes in neuroendocrine function	Changes in endocrine markers (Plasma, Saliva)	MEG, EEG (subcortical)	Cortisol level
Neuromuscular	Measures of molecular, cellular or physiological processes representative of changes in neuromuscular activation and function.	Changes in muscle tone, muscle activation	EMG	Max voluntary contraction, visualized muscle activation (US, fMRI, etc.)
Other mechanisms	Measures of molecular, cellular or physiological processes representative of changes not specified within the aforementioned domains.			

Definitions: MEG- magnetoencephalography, EEG- electroencephalogram, NCV- nerve conduction velocity, fMRI- functional magnetic resonance imaging, ANS- autonomic nervous system. US- Ultrasound
