# Supplementary material for: The mechanisms of manual therapy: A living review of systematic, narrative, and scoping reviews
Source: PLoS One. 2025 Mar 18;20(3):e0319586. doi: 10.1371/journal.pone.0319586 (PMC11918397; doi:10.1371/journal.pone.0319586)
Supplement: S5 Appendix — (PDF) [file pone.0319586.s005.pdf]

S4 Appendix: AMSTAR-2 Scores

Author(s):	Item #1	Item #2	Item #3	Item #4	Item #5	Item #6	Item #7	Item #8	Item #9	Item #10	Item #11	Item #12	Item #13	Item #14	Item #15	Item #16
Gera et al. 2020	Y	Y	Y	P	Y	Y	N	Y	Y	N	Y	Y	Y	Y	Y	Y
Bernier Carney et al. 2020	N	N	N	N	Y	N	N	N	P	N	N/A	N/A	N	N	N/A	Y
Voogt et al. 2015	Y	P	N	P	N	N	N	P	Y	N	N/A	N/A	N	N	N/A	N
Zegarra-Parodi et al. 2015	N	N	N	P	Y	Y	N	Y	N	Y	N/A	N/A	N	N	N/A	Y
Chow et al. 2021	N	P	Y	P	Y	N	N	P	Y	Y	N/A	N/A	Y	N	N/A	Y
Araujo et al. 2019	Y	Y	N	P	Y	Y	P	P	Y	N	N/A	N/A	Y	Y	N/A	Y
Sampath et al. 2017	Y	Y	Y	Y	Y	Y	Y	Y	Y	Y	Y	Y	Y	N	N	Y
Coronado et al. 2012	Y	P	Y	P	N	Y	N	P	Y	N	Y	N	N	N	Y	N
Sullivan et al. 2020	N	N	N	P	N	N	N	P	N	N	N/A	N/A	N	N	N/A	Y
Gay et al. 2013	Y	P	Y	P	N	Y	Y	P	Y	Y	Y	N	Y	N	N	Y
Souza et al. 2021	N	P	N	P	Y	N	N	N	Y	N	N/A	N/A	N	N	N/A	Y
Galindez-Ibarbengoetxea et al. 2017	Y	P	Y	P	Y	Y	N	Y	Y	N	N/A	N/A	N	N	N/A	Y
Navarro-Santana et al. 2020	Y	P	N	P	Y	Y	N	Y	Y	N	Y	Y	Y	Y	N	Y
Borges et al 2018	Y	P	Y	P	Y	N	N	Y	N	N	N/A	N/A	N	N	N/A	Y
Arribas-Romano et al. 2020	Y	P	Y	P	Y	Y	Y	Y	Y	N	Y	N	N	Y	Y	Y
Tejero-Fernandez	Y	P	N	P	Y	N	N	P	Y	N	N/A	N/A	Y	N	N/A	Y
Jones et al. 2013	Y	P	Y	P	N	N	N	Y	P	N	N/A	N/A	Y	N	N/A	Y
Rogan et al. 2022	N	P	Y	P	Y	Y	N	Y	N	N	N/A	N/A	N	N	N/A	Y
Xiong et al. 2015	Y	P	N	P	Y	Y	N	P	Y	N	Y	N	N	N	N	Y
Hillier et al. 2010	Y	Y	Y	Y	Y	Y	Y	Y	Y	N	Y	Y	Y	Y	N	Y
Nelson 2015	N	P	N	P	N	N	N	N	N	N	N/A	N/A	N	N	N/A	N
Lascurain-Aguirrebeña et al. 2016	N	P	N	P	N	N	N	P	Y	N	N/A	N/A	N	N	N/A	Y
Jung et al. 2023	Y	Y	N	P	Y	Y	Y	Y	Y	N	Y	N	Y	Y	N	Y
Schmid et al. 2008	N	P	N	P	N	N	N	P	Y	N	N	N	N	N	N	N
Chu et al. 2014	N	P	N	P	Y	N	N	P	Y	N	Y	Y	N	N	N	Y
Mitchell et al. 2017	N	P	N	P	Y	N	N	P	P	N	N/A	N/A	N	N	N/A	Y
Lima et al. 2020	N	P	N	P	Y	Y	N	N	N	N	N/A	N/A	N	N	N/A	N
Jun et al. 2020	N	P	N	P	Y	Y	N	N	N	N	N/A	N/A	N	Y	N/A	Y
Picchiottino et al. 2019	Y	P	Y	P	Y	Y	N	N	Y	N	Y	Y	Y	Y	N	Y
Millan et al. 2012	N	P	N	P	Y	Y	N	P	P	N	N/A	N/A	Y	N	N/A	Y
Corso et al. 2019	Y	P	Y	P	Y	N	N	Y	Y	N	N/A	N/A	Y	N	N/A	Y
Kingston et al. 2014	Y	P	N	P	Y	Y	Y	Y	Y	N	N/A	N/A	Y	N	N/A	N
Hegedus et al. 2011	N	P	N	N	Y	N	N	P	Y	N	N/A	N/A	N	N	N/A	N
Honoré et al. 2018	Y	P	N	P	N	Y	Y	P	P	N	N/A	N/A	Y	N	N/A	Y
Coronado et al. 2010	N	P	N	P	Y	N	N	P	Y	N	N/A	N/A	N	N	N/A	N
Meyer et al. 2019	Y	P	Y	P	N	N	N	P	P	N	N/A	N/A	Y	N	N/A	Y
Riley et al. 2024	Y	Y	N	Y	Y	N	Y	Y	Y	N	N/A	N/A	Y	Y	N/A	Y
Young et al. 2024	N	N	Y	PY	Y	Y	N	Y	Y	N	N/A	N/A	Y	N	N/A	Y
Sampath et al. 2024	Y	Y	Y	Y	Y	Y	Y	Y	Y	N	Y	Y	Y	Y	N	N
