# Supplementary material for: The mechanisms of manual therapy: A living review of systematic, narrative, and scoping reviews
Source: PLoS One. 2025 Mar 18;20(3):e0319586. doi: 10.1371/journal.pone.0319586 (PMC11918397; doi:10.1371/journal.pone.0319586)
Supplement: S6 Appendix — (PDF) [file pone.0319586.s006.pdf]

Appendix E: Risk of Bias (ROBIS) Scores for included Systematic and Scoping Reviews

Author	Item 1.1	Item 1.2	Item 1.3	Item 1.4	Item 1.5	Domain 1	Item 2.1	Item 2.2	Item 2.3	Item 2.4	Item 2.5	Domain 2	Item 3.1	Item 3.2	Item 3.3	Item 3.4	Item 3.5	Domain 3	Item 4.1	Item 4.2	Item 4.3	Item 4.4	Item 4.5	Item 4.6	Domain 4	Overall ROB 1	Overall ROB 2	Overall ROB 3	Risk of Bias
Gera et al. 2020	PY	PN	PN	PY	PN	HC	PY	Y	PY	PN	Y	UC	PY	PY	PY	Y	Y	LC	PY	PY	Y	Y	Y	Y	LC	Y	PY	PN	HIGH
Bernier Carney et al. 2020	PY	PY	PN	PY	PN	LC	PY	Y	N	N	Y	HC	PY	PY	PY	Y	Y	LC	PY	NI	NI	Y	NI	NI	UC	Y	Y	Y	LOW
Voogt et al. 2015	PY	Y	Y	PY	PY	LC	Y	Y	PY	N	N	HC	N	Y	PY	Y	Y	LC	PY	NI	PY	Y	PY	Y	LC	N	Y	Y	LOW
Zegarra-Parodi et al. 2015	PN	PN	PN	Y	Y	HC	Y	Y	Y	Y	Y	LC	PY	Y	Y	N	N	HC	PY	NI	PY	Y	PY	NI	HC	N	Y	Y	HIGH
Chow et al. 2021	Y	Y	Y	PY	PY	LC	Y	Y	Y	PN	Y	LC	Y	Y	PY	Y	Y	LC	PY	PY	Y	PY	PY	PY	LC	Y	Y	Y	LOW
Araujo et al. 2019	Y	Y	Y	Y	Y	LC	Y	Y	Y	Y	Y	LC	Y	Y	PY	Y	Y	LC	Y	Y	PY	PY	PY	PY	LC	Y	Y	PY	LOW
Kovanur-Sampath et al. 2017	Y	Y	Y	PY	PY	LC	Y	Y	Y	N	Y	LC	Y	Y	PY	Y	Y	LC	Y	Y	Y	Y	NI	Y	LC	Y	Y	Y	LOW
Coronado et al. 2012	PY	Y	Y	Y	PY	LC	Y	Y	Y	N	N	HC	Y	Y	PY	Y	Y	LC	PY	N	Y	Y	Y	Y	LC	Y	Y	Y	LOW
Sullivan et al. 2020	PN	NI	NI	NI	NI	HC	PN	NI	NI	NI	NI	HC	NI	PY	N	N	N	HC	NI	NI	NI	NI	NI	NI	HC	N	N	N	HIGH
Gay et al. 2013	PY	Y	Y	Y	PY	LC	PY	Y	Y	N	N	HC	Y	Y	Y	Y	Y	LC	Y	N	Y	PY	N	PY	LC	Y	Y	Y	LOW
Souza et al. 2021	PN	PN	PN	PN	PN	HC	PY	N	N	N	NI	HC	NI	N	N	Y	Y	HC	N	PN	N	N	N	N	HC	N	N	N	HIGH
Galindez-Ibarbengoetxea et al. 2017	PN	PN	PN	PY	PY	HC	Y	Y	PN	N	Y	HC	Y	Y	PN	Y	Y	LC	PY	NI	N	NI	PY	Y	HC	PN	PN	PY	HIGH
Navarro-Santana et al. 2020	Y	Y	Y	Y	Y	LC	Y	Y	Y	Y	Y	LC	Y	Y	Y	Y	Y	LC	Y	Y	Y	Y	PY	Y	LC	Y	Y	PY	LOW
Borges et al 2018	PN	PN	PN	NI	PN	HC	Y	N	PN	N	Y	HC	PN	N	N	Y	Y	HC	PY	N	N	N	N	N	HC	N	PN	N	HIGH
Arribas-Romano et al. 2020	Y	PY	PY	Y	Y	LC	Y	N	Y	Y	Y	LC	Y	Y	Y	Y	Y	LC	Y	Y	Y	Y	Y	Y	LC	Y	Y	Y	LOW
Tejero-Fernandez	PN	PY	PN	PN	PN	HC	Y	N	PN	N	Y	HC	PY	N	N	Y	Y	HC	PY	NI	N	N	N	N	HC	N	N	PN	HIGH
Jones et al. 2013	PY	Y	Y	PY	PN	LC	Y	Y	Y	PN	PN	HC	PN	Y	PN	Y	Y	LC	PY	PN	N	N	N	N	HC	N	PN	PN	HIGH
Rogan et al. 2022	PY	Y	Y	Y	Y	LC	Y	Y	Y	Y	Y	LC	Y	Y	PN	N	N	HC	PY	NI	N	N	N	N	HC	Y	PN	PN	HIGH
Xiong et al. 2015	PY	PY	PY	PY	PY	LC	Y	Y	PN	N	Y	HC	Y	Y	Y	N	Y	HC	Y	N	Y	Y	N	N	HC	PN	PN	PN	HIGH
Hillier et al. 2010	PY	Y	Y	Y	NI	LC	Y	Y	Y	NI	Y	LC	Y	Y	Y	Y	Y	LC	Y	Y	Y	Y	Y	Y	LC	Y	Y	Y	LOW
Nelson 2015	PN	PN	PN	PY	PY	HC	PN	PY	PN	N	N	HC	N	N	N	N	N	HC	PN	N	N	N	N	N	HC	N	N	N	HIGH
Lascurain-Aguirrebeña et al. 2016	PY	PY	PY	PY	Y	LC	Y	Y	Y	N	N	HC	N	PN	PY	Y	Y	LC	PY	NI	N	N	N	PY	HC	PN	Y	PN	HIGH
Jung et al. 2023	Y	Y	Y	Y	Y	LC	Y	N	Y	Y	Y	LC	Y	Y	Y	Y	Y	LC	Y	Y	Y	Y	Y	Y	LC	Y	Y	Y	LOW
Schmid et al. 2008	Y	PY	N	Y	Y	LC	Y	Y	Y	Y	PY	LC	PY	PY	Y	Y	PN	LC	Y	NI	Y	Y	N	Y	LC	Y	Y	Y	LOW
Chu et al. 2014	PY	Y	N	PN	PY	HC	Y	Y	PN	N	Y	HC	Y	Y	Y	Y	Y	LC	Y	NI	Y	Y	N	Y	LC	N	Y	Y	LOW
Mitchell et al. 2017	PY	PY	PY	PY	PY	LC	Y	Y	Y	N	PY	LC	Y	Y	Y	PY	PY	LC	PY	NI	N	N	N	PY	HC	PY	PY	PY	LOW
Lima et al. 2020	PN	NI	NI	NI	NI	HC	PY	Y	N	NI	N	HC	N	N	PN	N	N	HC	PN	N	Y	N	N	N	HC	N	N	N	HIGH
Jun et al. 2020	PY	Y	Y	PY	PY	LC	Y	Y	Y	N	Y	LC	Y	Y	Y	N	N	HC	Y	N	Y	N	N	N	HC	Y	Y	Y	LOW
Picchiottino et al. 2019	Y	Y	Y	Y	PN	LC	Y	Y	Y	N	Y	LC	Y	Y	Y	Y	Y	LC	Y	Y	Y	Y	Y	Y	LC	Y	Y	Y	LOW
Millan et al. 2012	PY	Y	Y	Y	Y	LC	PN	Y	Y	Y	PY	LC	Y	Y	Y	PN	Y	LC	Y	NI	PN	N	N	N	HC	Y	Y	Y	LOW
Corso et al. 2019	Y	Y	PY	Y	PY	LC	Y	Y	Y	N	Y	LC	Y	Y	Y	Y	Y	LC	Y	N	N	N	N	N	HC	Y	Y	Y	LOW
Kingston et al. 2014	PY	Y	Y	Y	PY	LC	Y	Y	NI	N	Y	LC	Y	Y	Y	Y	Y	LC	Y	NI	Y	N	N	Y	LC	Y	Y	Y	LOW
Hegedus et al. 2011	PY	Y	Y	Y	PY	LC	Y	Y	Y	PN	Y	LC	Y	Y	Y	Y	Y	LC	Y	N	Y	N	N	Y	HC	Y	Y	Y	LOW
Honoré et al. 2018	Y	PN	Y	PN	PY	HC	Y	Y	Y	N	Y	LC	Y	Y	Y	Y	NI	LC	Y	Y	N	N	N	N	HC	N	N	N	HIGH
Coronado et al. 2010	PY	Y	Y	Y	PY	LC	Y	Y	Y	N	Y	LC	Y	Y	Y	Y	Y	LC	Y	NI	Y	N	N	Y	HC	Y	Y	Y	LOW
Meyer et al. 2019	Y	Y	PY	PY	Y	LC	Y	Y	PY	PY	Y	LC	NI	Y	PY	Y	PY	LC	Y	Y	PY	N	N	NI	LC	Y	Y	Y	LOW
Riley et al. 2024	Y	Y	PY	PY	PY	LC	Y	N	Y	N	Y	HC	Y	Y	Y	Y	Y	LC	PY	Y	PN	Y	PN	Y	LC	N	Y	Y	LOW
Young et al. 2024	Y	Y	Y	Y	Y	LC	Y	N	Y	N	Y	UC	PY	Y	Y	Y	Y	LC	Y	PY	Y	Y	Y	Y	LC	Y	Y	Y	LOW
Sampath et al. 2024	Y	Y	Y	Y	PY	LC	PY	Y	Y	N	Y	LC	PY	Y	Y	Y	Y	LC	Y	PY	Y	Y	Y	Y	LC	Y	Y	PY	LOW

Definitions: Y- Yes, PY- Partial Yes, NI-= Not Included, PN- Partial No, N- No, LC- Low Concern, HC- High Concern
